# Ferric Iron/Shikonin Nanoparticle‐Embedded Hydrogels with Robust Adhesion and Healing Functions for Treating Oral Ulcers in Diabetes

**DOI:** 10.1002/advs.202405463

**Published:** 2024-10-11

**Authors:** Xiaojing Chen, Zhangping Li, XinXin Ge, Xiaoliang Qi, Yajing Xiang, Yizuo Shi, Ying Li, Yao Pan, Yingying Wang, Yiyu Ru, Kelei Huang, Jiatan Shao, Jianliang Shen, He Li

**Affiliations:** ^1^ Department of Otolaryngology The First Affiliated Hospital of Wenzhou Medical University Wenzhou Zhejiang 325000 China; ^2^ The Quzhou Affiliated Hospital of Wenzhou Medical University Quzhou People's Hospital Quzhou 324000 China; ^3^ School & Hospital of Stomatology Wenzhou Medical University Wenzhou Zhejiang 325027 China; ^4^ National Engineering Research Center of Ophthalmology and Optometry Eye Hospital Wenzhou Medical University Wenzhou Zhejiang 325027 China; ^5^ Zhejiang Engineering Research Center for Tissue Repair Materials Wenzhou Institute University of Chinese Academy of Sciences Wenzhou Zhejiang 325001 China

**Keywords:** diabetes, hydrogels, oral ulcers, shikonin, tissue adhesives

## Abstract

Oral ulcers can be addressed using various biomaterials designed to deliver medications or cytokines. Nevertheless, the effectiveness of these substances is frequently limited in many patients due to poor adherence, short retention time in the mouth, and less‐than‐optimal drug efficacy. In this study, a new hydrogel patch (FSH3) made of a silk fibroin/hyaluronic acid matrix with light‐sensitive adhesive qualities infused with ferric iron/shikonin nanoparticles to enhance healing effects is presented. Initially, this hydrogel forms an adhesive barrier over mucosal lesions through a straightforward local injection, solidifying when exposed to UV light. Subsequently, FSH3 demonstrates superior reactive oxygen species elimination and near‐infrared photothermal bactericidal activity. These characteristics support bacterial elimination and regulate oxidative levels, promoting a wound's progression from inflammation to tissue regeneration. In a diabetic rat model mimicking oral ulcers, FSH3 significantly speeds up healing by adjusting the inflammatory environment of the injured tissue, maintaining balance in oral microbiota, and promoting faster re‐epithelialization. Overall, the light‐sensitive FSH3 hydrogel shows potential for rapid wound recovery and may transform therapeutic methods for managing oral ulcers in diabetes.

## Graphical abstract

A novel light‐responsive hydrogel (FSH3) is crafted for treating diabetic oral ulcers. Upon application at the affected area, FSH3 binds robustly, delivering outstanding performance in eliminating reactive oxygen species and providing near‐infrared photothermal bactericidal effects. It successfully eradicates bacteria and adjusts the oxidative environment to enhance wound healing.

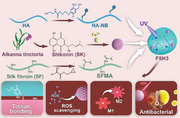

## 1 Introduction

Oral ulcers, often referred to as oral mucosal injuries, are a prevalent oral health issue characterized by continuous erosion or destruction of oral epithelial tissue. Worldwide, more than 25% of people have experienced or are experiencing oral ulcers.^[^ ^1^ ^]^ If not addressed promptly and effectively, the epithelial layer within these ulcers deteriorates, leading to depressions or even tissue necrosis.^[^ ^1^, ^2^ ^]^ This degradation significantly hampers essential functions like chewing, swallowing, speaking, and even digestion.^[^ ^3^ ^]^ Furthermore, oral lesions are susceptible to bacterial infections because of the diminished protective functions of the oral mucosa, which additionally hinders the ulcer healing process.^[^ ^4^ ^]^ Various therapeutic options have been formulated to speed up the recovery of oral ulcers, such as powders containing vitamins, commercial oral ulcer patches with flavonoids and chitosan, and ointments like growth factor gel.^[^ ^5^ ^]^ However, these treatments face challenges in efficiency, primarily because of their brief mucosal surface retention time in the dynamic and moist oral cavity environment, often under 2 h. Hence, developing biomaterials that possess superior adhesion characteristics is critical for improving the recovery of oral ulcers.

Crafting hydrogel patches with adhesion qualities offers a viable strategy for managing oral ulcers.^[^ ^6^ ^]^ Typically, the adhesion techniques of such patches are divided into two categories: physical and chemical adhesion, with many relying on a shift from liquid to solid to secure adhesion.^[^ ^7^ ^]^ Physical adhesion frequently employs hydrogen bonds, but achieving robust adhesion in a moist oral environment is challenging due to frequent failures.^[^ ^8^ ^]^ On the other hand, adhesion approaches that create chemical bonds with tissue surfaces offer stronger adhesion and aid cell migration at the adhesion site.^[^ ^9^ ^]^ Recent studies highlight a hydrogel composed of o‐nitrosobenzaldehyde (NB) grafted hyaluronic acid and double‐bond modified gelatin. This hydrogel stands out for its potent wet tissue adhesion, low swelling, swift gelation, and impressive biocompatibility—qualities crucial for applications in the saliva‐rich oral cavity.^[^ ^10^ ^]^ Furthermore, the hydrogel's primary ingredients, gelatin and hyaluronic acid, mirror the composition of the extracellular matrix (ECM) of oral mucosa, which is mainly proteins and polysaccharides.^[^ ^11^ ^]^ Consequently, this ECM‐mimicking adhesion hydrogel could be an optimal choice for hastening the healing of oral mucosal injuries.

Once the adhesive characteristics are attained, it is imperative for the hydrogel dressings to efficiently execute their designated biological roles, as this is key to ensuring superior repair of wounds resulting from oral ulcers.^[^ ^12^ ^]^ Healing of oral ulcers, similar to skin wound recovery, usually encompasses three intertwined phases: inflammation, proliferation, and remodeling.^[^ ^13^ ^]^ In repairing oral mucosa, a mucoadhesive hydrogel that autonomously regulates the wound environment—controlling bacterial contamination, inflammation, and host cell activity—and protects the injury site within a salivary setting for a prolonged duration (ideally exceeding 12 h, deemed optimal for oral mucosal treatments) is vital for supporting the regeneration of the oral mucosa.^[^ ^14^ ^]^ Recently, a range of bioactive materials has been incorporated into hydrogels for skin wound healing. These materials are recognized for their ability to accelerate tissue regeneration via anti‐inflammatory effects, sterilization, and the promotion of growth factor production, all without the necessity of adding cytokines, cells, or drugs.^[^ ^15^ ^]^ Nevertheless, these materials frequently exhibit insufficient adhesive strength and stability in moist environments. Furthermore, their complex architectural design, functional alterations, and the need for outside interventions can impede their application in clinical environments. Consequently, the development of oral ulcer patches that provide robust adhesion along with effective biological healing capabilities is critically necessary.

**Scheme 1:**
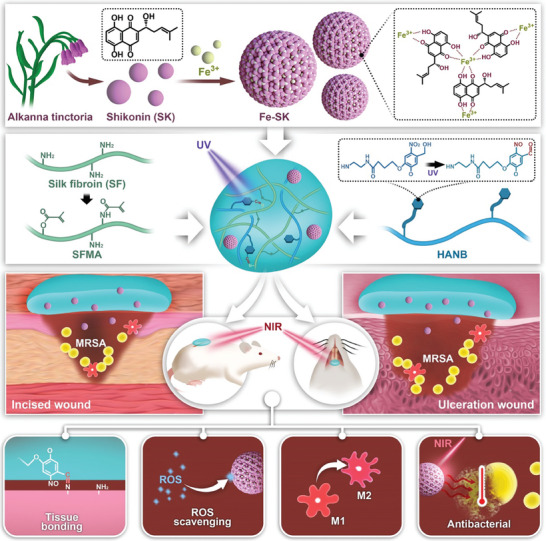
Depiction of the creation and utilization of FSH3 hydrogel for expedited healing of bacterial infections in wounds of diabetic rats.

This research focuses on creating an effective treatment for oral ulcers by developing an ECM‐like hydrogel patch (abbreviated to FSH3, **Scheme** **1**). The dressing incorporates ferric iron/shikonin nanoparticles (recognized for their therapeutic benefits) into a biomolecular matrix comprising methacrylate silk fibroin (SFMA) and NB‐grafted hyaluronic acid (HA‐NB), which react to ultraviolet (UV) light for curing and adhesion. When exposed to 365 nm UV light, the hydrogel creates a protective layer over the wound by undergoing radical polymerization. Additionally, the FSH3 hydrogel shows strong adhesion to wet tissues because the o‐nitrobenzene molecule in HA‐NB converts into an aldehyde molecule under UV light illumination, promoting rapid and strong adhesion to tissue by forming imine bonds interactions with the amino groups present on the tissue surface.^[^ ^3^ ^]^ Moreover, the ferric iron/shikonin nanoparticles embedded in the FSH3 hydrogel demonstrate outstanding near‐infrared photothermal germicidal properties and the ability to eliminate reactive oxygen species. These features are instrumental in bacterial eradication and oxidation state regulation, thus aiding the wound in progressing from an inflammatory stage to a proliferative one. The 3‐D porous architecture of FSH3, made from derivatives of natural materials, serves as an ideal scaffold for cells, promoting cell adhesion, growth, and angiogenesis throughout tissue remodeling. The efficacy of FSH3 in enhancing wound repair was evidenced in a streptozotocin‐administered bacterial‐infected rat model.

## 2 Results and Discussion

### 2.1 Synthesis and Adhesive Performance of Prepared Hydrogels

**Figure 1:**
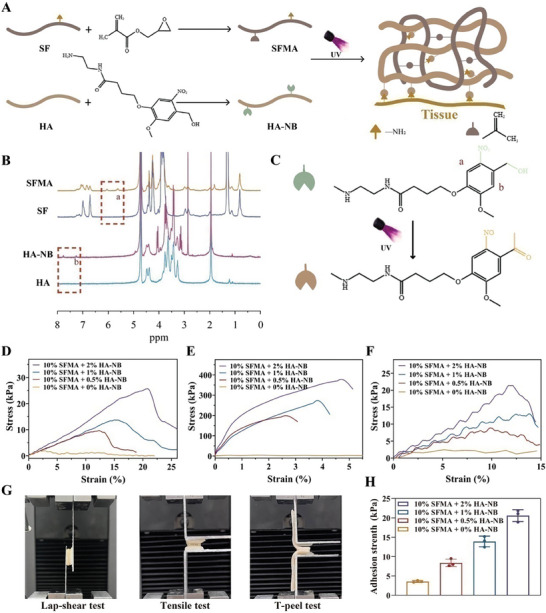
Synthesis of matrix hydrogels and evaluation of their adhesive qualities. A) Formation of SFMA, HA‐NB, and the corresponding hydrogels. B) Depiction of ^1^H NMR spectra for HA, HA‐NB, SF, and SFMA. C) Illustrative representation of the photo‐induced imine‐crosslinked hydrogel synthesis. D–F) Display of lap‐shear (D), tensile (E), and t‐peel (F) adhesion testing curves for hydrogels varying in HA‐NB levels. G) Overview of testing procedures: lap‐shear, tensile adhesion, and t‐peel tests. H) Results from the t‐peel test using hydrogels with diverse concentrations of HA‐NB. Error bars represent mean ± SD (n = 3).

In **Figure** **1**A, the interaction between the amino group of Silk fibroin (SF) and glycidyl methacrylate, resulting in SFMA synthesis, is depicted. Concurrently, the amino group of NB (─NH_2_) bonds with HA's carboxyl group (─COOH) through an amide bond to create HA‐NB. The successful grafting of methacrylate groups onto SF molecular chains is evidenced in the ^1^H NMR spectrum of Figure 1B. Calculations indicate that the SF methacrylate degree is ≈11.2%. The ^1^H NMR spectrum for HA‐NB shows peaks at δ = 7.67 ppm and δ = 7.32 ppm, which are aligned with protons at the a, b position on NB, confirming the successful attachment of o‐nitroso benzaldehyde groups to HA.^[^ ^3^ ^]^ The grafting degree of NB is ≈0.94%.^[^ ^16^ ^]^ Furthermore, Figure 1C illustrates the process leading to the formation of a hydrogel on the skin by the reaction of HA‐NB and SFMA. When exposed to UV light, the double bonds in SFMA undergo polymerization, creating a cross‐linked hydrogel network. Simultaneously, UV exposure generates aldehyde groups on HA‐NB, which then form dynamic covalent bonds with SFMA's amino moieties and the skin. FTIR spectroscopy analysis (Figure S1, Supporting Information) revealed specific absorption peaks at 1638 cm^−1^ and 1520 cm^−1^ for SFMA, characteristic of ─CH_2_═CH_2_─, and at 1612 and 1404 cm^−1^ for HA‐NB, indicating ─NB bending vibrations. Additionally, a marked increase in absorption bands between 500 and 800 cm^−1^, indicative of C═O vibrations, confirmed the successful synthesis of the FSH hydrogels.

Optimal adhesive materials enhance tissue integration and regeneration in pathophysiological environments.^[^ ^17^ ^]^ The superior adhesion of hydrogels aids in maintaining contact with the target tissue, fostering better integration and healing. The adhesive qualities of hydrogels with varying HA‐NB concentrations were assessed through shear strength, tensile strength, and interfacial toughness tests between two wet tissues, following the standards of the American Society for Testing Material (ASTM) (Figure 1G). Hydrogels with varying HA‐NB concentrations displayed greater lap‐shear strength compared to the pure SFMA group (Figure 1D).^[^ ^18^ ^]^ During the tensile test depicted in Figure 1E, hydrogels with various HA‐NB concentrations demonstrated adhesion strengths 76, 55, and 40 times greater than the pure SFMA group. Similarly, T‐peel test results showed enhanced interface toughness for these hydrogels (20.59 ± 1.52 kPa for 10% SFMA + 2% HA‐NB and 13.9 ± 1.37 kPa for 10% SFMA + 1% HA‐NB), which was 5.8 and 3.9 times stronger compared to the 10% SFMA with no HA‐NB (3.56 ± 0.23 kPa), as shown in Figure 1F,H. Overall, hydrogels incorporating HA‐NB outperformed those with only SFMA in mechanical tests, suggesting their potential as effective clinical adhesives.

Following comprehensive optimization and characterization investigations into the chemical composition and adhesive abilities of the developed hydrogels, we selected a combination of 10% SFMA and 1% HA‐NB, including 0.4% of the polymerization photoinitiator [lithium phenyl(2,4,6‐trimethylbenzoyl)phosphinate, LAP], for subsequent experiments.

### 2.2 Synthesis and Characterization of FeSK Nanoparticles

**Figure 2:**
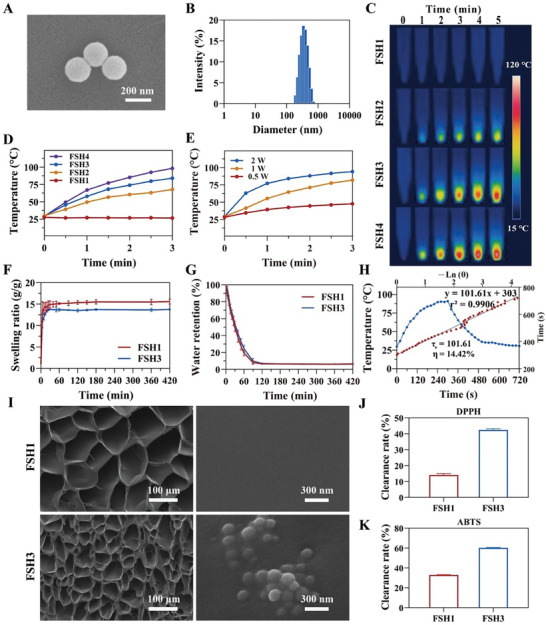
Analysis of FeSK nanoparticles' physiochemical properties and FSH hydrogels. A,B) SEM imaging (A) and size distribution (B) of FeSK nanoparticles. C,D) Thermal imagery (C) and graphs showing temperature changes (D) in FSH hydrogels. E) Profile of temperature variation in FSH3 hydrogel under different near‐infrared (NIR) irradiation scenarios. F,G) Assessment of swelling (F) and moisture retention capacity (G) in FSH hydrogels. H) Graph illustrating cooling duration in relation to the inverse natural logarithm of the temperature drive during FeSK's cooling stage following 808 nm NIR irradiation (photothermal efficiency = 14.42%). I) SEM images showcasing FSH1 and FSH3 at varied magnifications. J,K) DPPH (J) and ABTS (K) scavenged by FSH1 and FSH3 hydrogels. Error bars represent mean ± SD (n = 3).

The morphological features of FeSK are shown in **Figure** **2**A through scanning electron microscope (SEM) imaging. FeSK nanoparticles exhibited uniformity in shape, appearing as distinct nanospheres without visible clustering. Dynamic light scattering data complemented this observation, indicating an average nanoparticle size of 356.8 ± 9.4 nm (Figure 2B). Furthermore, the FeSK nanoparticles demonstrated a zeta potential value of −34.6 mV, illustrated in Figure S2 (Supporting Information), potentially attributable to polyphenolic groups on the FeSK's surface.

Next, the photothermal characteristics of FeSK under NIR light were methodically investigated. We initiated the experiment by exposing different concentrations of FeSK to 808 nm laser radiation at 1 W cm^−2^. During this, an infrared camera captured real‐time thermal images of FeSK (Figure S3, Supporting Information). Notably, FeSK exhibited a significantly higher temperature increase compared to phosphate buffer saline (PBS). Furthermore, the photothermal efficiency of FeSK was determined to be 14.42% (referenced in Figure 2H; Figure S4, Supporting Information). These findings highlight FeSK nanoparticles' exceptional photothermal stability and efficiency, suggesting their strong potential in photothermal antibacterial applications.

### 2.3 Synthesis and Characterization of FSH Hydrogels

Figure 2D illustrates how the FSH hydrogels' temperature swiftly escalated with higher FeSK concentrations. Particularly, a hydrogel containing 0.5 mg mL^−1^ FeSK reached 60.4 °C, while at 1 and 2 mg mL^−1^ concentrations, the temperatures attained were 73.9 and 85.2 °C after 2 min of irradiation. Increasing the laser power density also resulted in elevated temperatures in FSH3 hydrogels with a fixed concentration, as shown in Figure 2E. Additionally, the thermal image captured after 5 min of 808 nm irradiation aligned with the temperature escalation curve (Figure 2C). The photostability of the hydrogel was assessed by allowing it to return to ambient temperature under natural light for ≈7 min. This stability was confirmed, as indicated in Figure S5 (Supporting Information), by the sustained temperature rise even after four cycles of laser exposure, underscoring the hydrogel's remarkable photothermal resilience.

Subsequently, thermogravimetric analysis (TGA) was conducted in an air environment from 50 to 600 °C to assess the thermal stability of the newly formulated FSH hydrogels. Figure S6 illustrates that the specimens exhibited three distinct decomposition temperatures. Initially, the weight of all specimens gradually decreased below 200 °C due to the evaporation of free water from the specimens. In the next stage, the hydrogel's weight decreased between 200 and 400 °C due to the breakdown of the polymer side chain groups. Beyond 400 °C, the hydrogel began its oxidation, decomposition, and charring process resulting from the main polysaccharide chain's fragmentation. At 600 °C, the total mass loss rates for FSH1 and FSH3 hydrogels were 63.5% and 59.5%, respectively, suggesting that the inclusion of FeSK nanoparticles within the polysaccharide matrix enhanced the hydrogel scaffold's stability.

After that, the FSH hydrogels underwent an assessment for their swelling and deswelling properties. Observations from the swelling curves (Figure 2F) revealed that the water uptake patterns of FSH1 and FSH3 hydrogels were similar, exhibiting quick water absorption in the initial 3 min and then leveling off after 30 min. Upon reaching equilibrium, FSH1 displayed a higher swelling ratio (16.3) compared to FSH3 (14.1), indicating enhanced cross‐linking in the hydrogels post‐FeSK addition. During the water retention test, FSH hydrogels were dried at 37 °C until their weight reached stability. FSH3 demonstrated marginally superior water retention than FSH1 (Figure 2G). These findings indicate that FSH hydrogels have outstanding water absorption and retention properties, enabling them to quickly soak up wound exudate and sustain an appropriate moist environment for wound healing.^[^ ^19^ ^]^

Next, a SEM analysis was conducted to observe the surface texture and micro‐pore arrangement of the freeze‐dried hydrogels. The images displayed in Figure 2I revealed that the porous network structure in both FSH1 and FSH3 created an ideal setting for absorbing exudate and promoting cell proliferation. Higher magnification SEM views indicated a smooth internal texture in FSH1, whereas FSH3 exhibited a rougher interior due to FeSK nanoparticles. Intriguingly, the inclusion of FeSK led to average pore sizes in FSH1 and FSH3, measuring 62.5 µm and 38.5 µm, respectively.

Besides, we explored the antioxidant capabilities of FSH hydrogels. High reactive oxygen species (ROS) levels in wounds can substantially impede the healing process. Our approach involved utilizing 1,1‐Diphenyl‐2‐picrylhydrazyl (DPPH) and 2,2ʹ‐azino‐bis(3‐ethylbenzothiazoline‐6‐sulfonic acid) diammonium salt (ABTS) radicals to assess the hydrogels' capacity for neutralizing free radicals. Initially, DPPH was incubated with FSH hydrogels for half an hour. Notably, the FSH3 hydrogel, which contains FeSK, demonstrated enhanced scavenging efficacy compared to FSH1, thus verifying its superior antioxidant effectiveness. The scavenging percentages for FSH1 and FSH3 were recorded at 14.0% and 42.5%, respectively, as shown in Figure 2J. Furthermore, further validation of the antioxidant characteristics of these hydrogels was performed through tests with ABTS radicals, as depicted in Figure 2K. Consistent with our expectations, the ABTS assay outcomes mirrored those observed with DPPH. Consequently, the FSH3 hydrogel we developed showcased a commendable ability to scavenge radicals, suggesting its potential to facilitate antioxidant processes during wound healing.

### 2.4 In Vitro Biocompatibility and Antioxidant Properties of FSH Hydrogels

**Figure 3:**
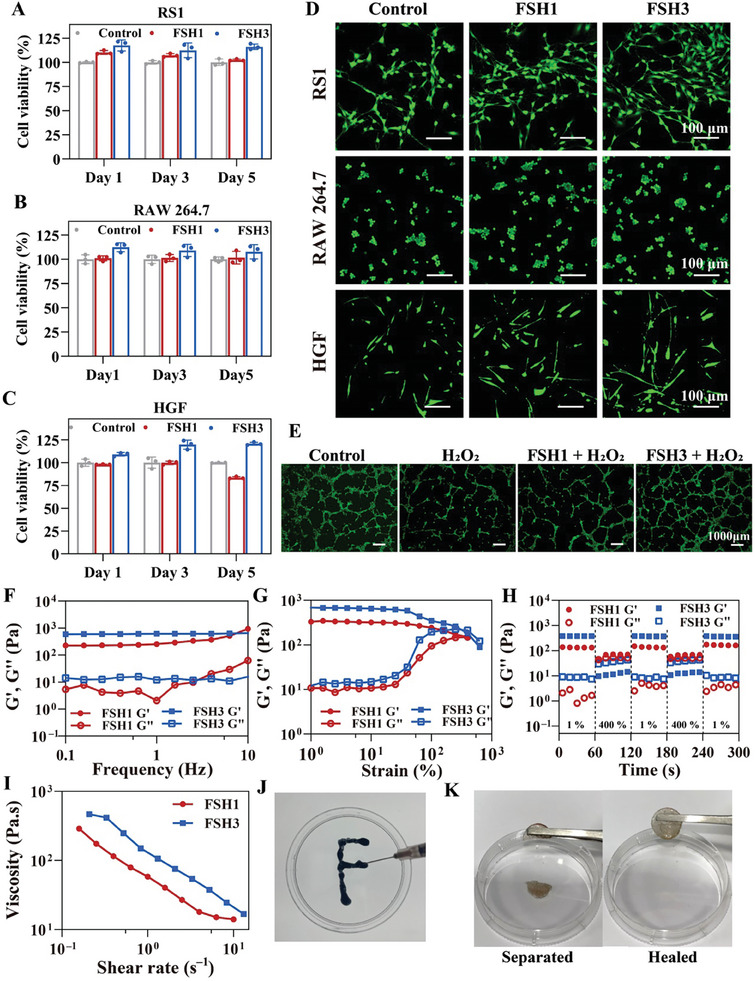
Analyzing the mechanics and antioxidant capabilities of FSH hydrogels. A–C) CCK‐8 method assessment of cytotoxic effects exerted by FSH hydrogels on RS1 (A), RAW 264.7 (B), and HGF (C) cells. D) Outcomes of calcein‐AM/PI for tested cells after 72 h of exposure to various treatments. E) Representative images of in vitro tube formation. F–I) Analysis of the rheological characteristics of FSH, including frequency sweeps (F), strain amplitude sweeps (G), dynamic strain steps (H), and shear‐thinning behavior measurements (I). J) Images depicting the application of FSH3 hydrogel on an artificial disc. K) Photographs highlighting the self‐healing properties of the FSH3 hydrogel. Error bars represent mean ± SD (n = 3).

We further evaluated the FSH hydrogels performance at a cellular level. The safety of these materials is critical for their application in tissue engineering and wound healing as versatile dressings. Therefore, we initially investigated the cytocompatibility of the developed FSH hydrogels. The Cell Counting Kit (CCK)‐8 experiment was adopted to evaluate cell viability following incubation of RS1, RAW 264.7, and HGF cells with FSH hydrogel extracts over periods of 1, 3, and 5 days. **Figure** **3**A–C show that the cell viability in FSH1 and FSH3 sets exceeded 100%, indicating negligible toxicity of the hydrogels.^[^ ^20^ ^]^ RS1, RAW 264.7, and HGF cells were stained with the cell live/dead probe after 3 days of cell culture. The green fluorescence observed in the FSH set was similar to that in the control set, with a minimal number of dead cells noted (Figure 3D). Next, the hemocompatibility of the FSH hydrogel was tested through a hemolysis assay. Figure S7 (Supporting Information) shows that the double distilled water (DDW) supernatant appeared bright red, which indicates that RBCs are lysed and, subsequently, hemoglobin is released. Conversely, the supernatants from both the PBS and hydrogel groups remained transparent, with a hemolysis rate of less than 5%, confirming their excellent hemocompatibility.^[^ ^21^ ^]^ In summary, the FSH hydrogels demonstrated outstanding biocompatibility, rendering them ideal as scaffold materials for biomedical applications.

Furthermore, HUVEC cells were utilized to assess the effects of newly created FSH hydrogels on angiogenesis in conditions characterized by oxidative stress.^[^ ^22^ ^]^ These cells were distributed across the Matrigel following their stay in the incubator. As the cultivation period reached 8 h, within the control group, HUVEC cells interconnected to form a network of tubes, distinguished by a higher count of blood vessels and improved structural integrity (Figure 3E). Hydrogen peroxide (H_2_O_2_) markedly suppressed angiogenic activity in HUVEC cells, leading to an absence of angiogenesis. Conversely, cells in the FSH3 + H_2_O_2_ group showed closeness and the potential to form a tubular network. This suggested that the FSH hydrogel effectively countered the detrimental effects of ROS, preserving the angiogenic capabilities of HUVCE cells.

### 2.5 Mechanical and Antioxidant Properties of FSH Hydrogels

Hydrogel dressings require adequate mechanical performance and flexibility to conform to the dynamic and intricate wound environment.^[^ ^23^ ^]^ The mechanical characteristics of FSH hydrogels were extensively analyzed employing a DHR‐2 rheometer. Initially, oscillation frequency tests from 0.1 to 10 Hz assessed the hydrogels frequency‐dependent rheological responses (Figure 3F), revealing stable periods during which the storage modulus (G') consistently exceeded the loss modulus (G″).^[^ ^24^ ^]^ Afterward, strain scan tests were conducted to analyze the linear range of viscoelasticity of the FSH hydrogels (Figure 3G). The FSH3 exhibited a transition point ≈100%, signifying a colloidal state upon strain surpassing 100%. Subsequently, amplitude tests were performed to assess the self‐healing performance of the FSH specimens. Figure 3H illustrates the effects of high strain (400%) on the network, followed by the assessment of recovery at low strain (1%). At 400% strain, a significant decrease in G' below G″ indicated a fracture in the hydrogel network. When the strain was returned to 1%, G' and G″ returned to their initial values, demonstrating the rapid self‐healing of the hydrogel.^[^ ^25^ ^]^ Repeated cycles of network recovery and collapse demonstrated the impressive self‐healing properties of the hydrogel, attributed to dynamic reversible cross‐linking from Schiff base reactions between polymer chains in the FSH3 hydrogel. Lastly, a shearing test indicated shear‐thinning properties due to dynamic physical cross‐links in the network, highlighting the hydrogel's effective injectability (Figure 3I). Figure S8 (Supporting Information) displays significant enhancement in the gel's mechanical properties following exposure to ultraviolet light, with the G' increasing roughly a hundred times from 10 to 1000 Pa. Additionally, the FSH hydrogels demonstrated their injectability and self‐repairing features, as confirmed by macroscopic photography. Strips of hydrogel were smoothly extruded into a glass container containing PBS using a 5 mL syringe (Figure S9, Supporting Information). By injecting the hydrogel into a dish to form the letter “F”, we showcased the hydrogel's proficient injection capabilities (Figure 3J). Following the division of the FSH3 hydrogel into two parts, these segments naturally rejoined within 2 h, exhibiting minimal deviation from their original form (Figure 3K). Such findings indicate that FSH hydrogels have advanced mechanical properties, especially in terms of injectability and self‐healing, which make them ideal for managing complex wound environments.^[^ ^26^ ^]^ In contrast to conventional dressings that might detach or not adhere well to wounds, FSH3 hydrogel demonstrated robust attachment to various tissues in rats (Figure S10, Supporting Information). This strong adhesion underpins its antimicrobial, anti‐inflammatory, and wound‐healing capabilities.^[^ ^27^ ^]^

### 2.6 Assay for Cell Migration and Angiogenesis In Vitro

**Figure 4:**
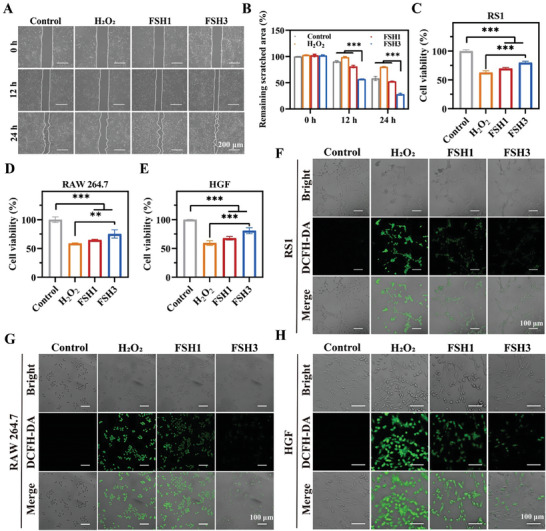
Cellular studies in vitro using FSH hydrogels. A) Migration analysis of RS1 cells following a scratch at 0, 12, and 24 h (scale bar representing 200 µm). B) Remaining area from the scratch test. C–E) Cell viability assessment for RS1 (C), RAW 264.7 (D), and HGF (E) cultures in H_2_O_2_‐enriched medium with different hydrogel formulations. F–H) Fluorescent imaging of RS1 cells (F), RAW 264.7 macrophages (G), and HGF cells (H) after exposure to H_2_O_2_ in different sets. Error bars represent mean ± SD (n = 3). *** signifies *p* < 0.001.

We utilized cell scratch assays to assess FSH hydrogels impact on cellular migration under conditions of oxidative stress. A pipette tip was used to create a scratch on the bottom of a plate with RS1 cells. After adding H_2_O_2_ and hydrogel, we observed changes in the scratch zone over 12 and 24 h of incubation. **Figure** **4**A illustrates that over time, in the control set, cells migrated and proliferated effectively, significantly shortening the migration distance within 24 h. In contrast, the scratch area in the H_2_O_2_ set showed little change, with cells barely migrating into the scratched region. Cells treated with FSH3 exhibited enhanced migratory abilities relative to the H_2_O_2_ set, resulting in a progressive reduction in the scratched surface area. The last remaining scratch area amounted to just 27.25% of the original, as shown in Figure 4B. These findings indicate that the synthesized hydrogel boosts cells antioxidant stress response by neutralizing exogenous oxidants.

Moreover, the hydrogel's ability to act as an antioxidant was verified at the cellular scale. Figure 4C–E depicts that, following treatment with H_2_O_2_, oxidative damage significantly suppressed cell proliferation. As anticipated, cells exposed to the FSH hydrogels displayed a marked improvement in survival rates. Specifically, when RS1, RAW 264.7 and HGF cells were incubated with both 700 µm H_2_O_2_ and the hydrogel for 4 h, the survival rates observed were 79.8% for RS1, 75.1% for RAW 264.7 and 80.8% for HGF, compared to the untreated control. These findings indicate that FSH3 hydrogel effectively neutralizes ROS.

Furthermore, cells underwent staining using the DCFH‐DA marker to assess intracellular ROS concentrations, aiming to explore hydrogel's defensive capabilities amidst oxidative stress.^[^ ^28^ ^]^ Confocal fluorescence imaging (Figure 4F–H) revealed enhanced fluorescence intensity and augmented green fluorescence in RS1 cells, HGF cells, and RAW 264.7 macrophages within the H_2_O_2_ set, signifying elevated ROS levels. Conversely, the FSH3 hydrogel cohort exhibited markedly diminished green fluorescence, denoting reduced ROS quantities. Such findings underscore the remarkable antioxidative capability of the FSH hydrogel, which incorporates FeSK, to safeguard cells from oxidative stress, underscoring its viability for applications in healing chronic wounds.

### 2.7 Evaluating FSH Hydrogels Photothermal Antibacterial Efficacy In Vitro

**Figure 5:**
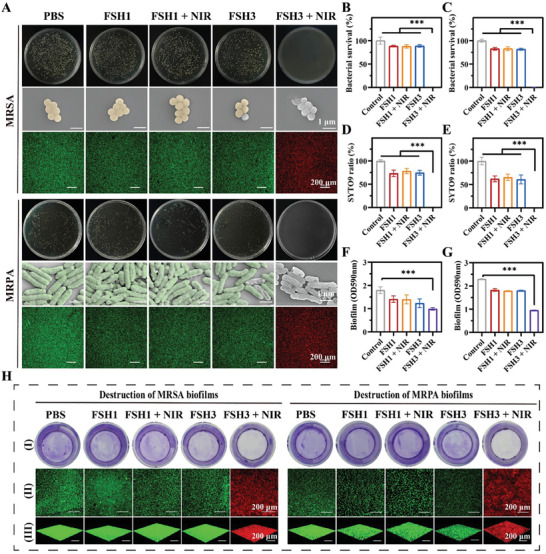
Evaluating the in vitro photothermal antibacterial performance of FSH samples. A) Analysis of antibacterial activity against MRSA and MRPA employing techniques including enumeration, SEM imaging, and fluorescence staining. B,C) Survival rates of MRSA (B) and MRPA (C) in various test samples. D,E) Comparative SYTO9 fluorescence intensity under different experimental setups for MRSA (D) and MRPA (E). F,G) Measurement of biofilm mass for MRSA (F) and MRPA (G). H) Breakdown of established MRSA and MRPA biofilms by various samples, analyzed using crystal violet staining (I), 2D staining (II), and 3D staining (III). Error bars represent mean ± SD (n = 3). *** signifies *p* < 0.001.

Infection significantly impedes the full healing of wounds.^[^ ^29^ ^]^ In this work, we initially utilized the agar plate counting methodology to investigate the antibacterial efficacy of FSH systems. Antibacterial tests against gram‐positive (MRSA) and gram‐negative (MRPA) bacteria were performed with and without 808 nm NIR laser exposure (**Figure** **5**A). The hydrogel group managed to adhere to some bacteria, achieving antibacterial efficacy at a rate of 14% for MRSA (Figure 5B) and 18% for MRPA (Figure 5C). As expected, the FSH3 + NIR set exhibited an antibacterial efficacy close to 100%, suggesting that the use of the NIR laser (808 nm, 1 W cm^−2^, for 5 min) notably augmented the FSH3 hydrogel's sterilizing capability.

A live/dead bacterial staining experiment was conducted to examine the inhibitory effect of FSH hydrogel on bacterial growth (Figure 5A). Groups treated with FSH1, FSH3, and FSH1 + NIR exhibited diminished green fluorescence compared to the control set, indicating the hydrogel's capacity to ensnare bacteria (Figure 5D,E). Notably, the FSH3 + NIR group demonstrated the most intense red fluorescence. This is due to the permeation of PI into damaged bacterial membranes, coloring the bacteria red. PI fluorescence intensity of MRSA and MRPA under different experimental setups is demonstrated in Figures S11 and S12 (Supporting Information).

To elucidate the antibacterial strategy more clearly, we analyzed the bacterial membrane's structure post‐treatment using SEM techniques.^[^ ^30^ ^]^ SEM imagery indicated that MRSA and MRPA bacteria within the control group maintained their morphological integrity, featuring smooth membranes.^[^ ^31^ ^]^ Intriguingly, bacteria within the FSH3 hydrogel cohort exhibited compromised structural integrity, with cell walls and membranes appearing wrinkled.^[^ ^32^ ^]^ This effect is attributed to the hydrogels modifying the bacterial membrane potential and inhibiting metabolic activity via electrostatic adsorption. Bacteria exposed to both FSH3 hydrogel and NIR laser experienced more severe damage, including cell membrane breaches or even complete perforations. These findings validate the bactericidal capability of the FSH3 hydrogel, enhanced by the photothermal action initiated by NIR exposure. It is believed that the effective bacterial entrapment combined with the NIR‐induced photothermal response of the FSH3 hydrogel plays a significant role in boosting its antibacterial efficacy. Overall, the FSH3 hydrogel demonstrates remarkable photothermal antibacterial properties and serves as a versatile hydrogel dressing for wounds, offering both antibacterial and antioxidant benefits. Quantitative plate counts revealed that the combination of FSH3 with NIR eradicated 100% of MRSA and 94.25% of MRPA bacteria, outperforming all other tested groups.

Treating chronic wounds becomes significantly complicated when faced with biofilms resistant to antibiotics.^[^ ^33^ ^]^ These biofilms, self‐organized matrices filled with water and extracellular polymers, resist conventional antibiotic strategies.^[^ ^32^ ^]^ Demonstrations show that FSH3 + NIR effectively disrupts MRSA and MRPA biofilms, achieving a 99% dissolution rate, as seen in Figure 5. Our exploration into MRSA and MRPA biofilms involved crystal violet staining, with confirmation through both 3‐D and 2‐D examinations via laser scanning confocal microscopy. Fluorescence intensity ratios, indicative of biofilm disruption, are depicted in Figures S13 and S14 (Supporting Information). These results underscore (FSH3 + NIR)’s potent antimicrobial and biofilm‐disrupting properties, positioning it as an effective strategy against chronic wounds plagued by antibiotic‐resistant infections.^[^ ^34^ ^]^

### 2.8 In Vivo Healing Efficacy of FSH3 Hydrogel on MRSA‐Infected Diabetic Dorsal Skin Wounds

After obtaining in vitro data, we then assessed the potential of the FSH3 hydrogel for diabetic wound healing in vivo. Initially, the focus was on assessing the hemostatic capability of FSH3 hydrogels. Rapid hemostasis is crucial for quality repair when a wound occurs. Consequently, in our study, NB groups with light‐activated adhesive properties were incorporated into the gel matrix, targeting swift hemostasis with hydrogel dressings. The hemostatic effectiveness of FSH3 hydrogel was estimated by adopting rat liver and tail hemostasis models.^[^ ^35^ ^]^ In the hepatic hemostasis model (Figure S15, Supporting Information), rats treated with the FSH3 demonstrated the most effective hemostasis with minimal visible blood, in contrast to notable bleeding in other groups. Quantitative analysis showed significantly higher blood loss in the blank (199.2 mg) and gauze (132.7 mg) groups compared to the FSH3 hydrogel set (11.4 mg). The tail hemostasis model (Figure S16, Supporting Information) yielded similar findings, further affirming the superior bleeding control provided by FSH3 hydrogel in vivo.

**Figure 6:**
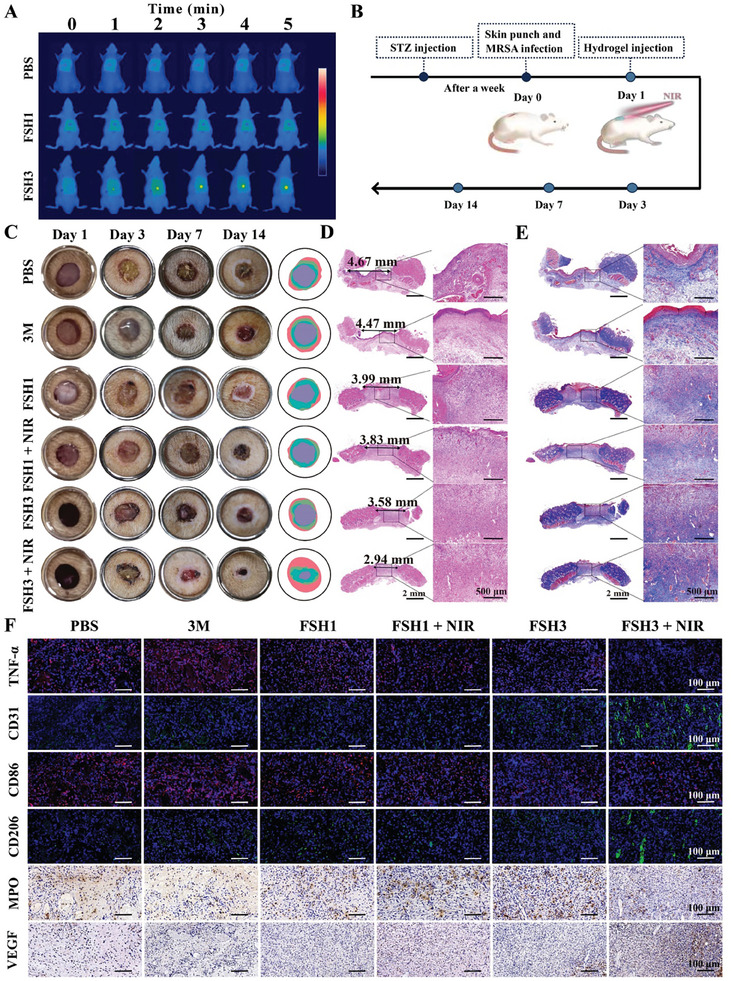
Assessment of the therapeutic efficacy of FSH3 hydrogel on MRSA‐infected dorsal skin lesions in diabetic rats. A) Infrared thermal images of rat wound sites after various treatments. B) Schematic representation of the infected lesion creation and its healing path. C) Representative healing process images of diabetic rat wounds under various treatments, including the extent of wound closure achieved with different therapies on days 1, 3, 7, and 14. D) Day 14 histopathological H&E stain of the lesion area along with detailed images of H&E‐stained sections. E) Masson's trichrome stain at the lesion site on day 14, including magnified views of Masson‐stained sections. F) Immunohistochemical staining for TNF‐α, CD31, CD86, CD206, MPO, and VEGF in the lesion area on day 7.

Then, the abilities of FSH3 hydrogel for photothermal heating and sterilization were assessed. **Figure** **6**A demonstrates that after 5 min of NIR irradiation, the temperatures at the rat wound regions of PBS‐ and FSH1‐treated rats exhibited minimal fluctuation. However, in the FSH3 + NIR group, the temperature quickly escalated from 35.3 to 52.6 °C. Following different interventions, bacteria were gathered from the wounds and identified for quantification by adopting the agar plate approach (Figure S17, Supporting Information). Corresponding to the hydrogel in vitro antimicrobial properties, the highest bacterial levels were observed in control and 3 m sets, with FSH1 and FSH3 groups displaying moderate bacterial counts and the FSH3 + NIR set showing the least. These findings validate that the FSH3 hydrogel, particularly when used with NIR, offers superior antibacterial efficacy. Temperatures reaching 52.6 °C prove capable of eradicating bacteria in wounds, yet the repercussions on nearby healthy skin tissue must be considered. To explore the high temperature effects on normal skin systematically, rat skin was subjected to heat for 3 min using thermostatic soldering irons at temperatures of 40, 55, and 70 °C, with damage being recorded at each temperature setting. Documentation of the effects (Figure S18, Supporting Information) revealed a minor red scald mark on the rat skin at 55 °C, indicating some cellular damage. Four days later, the mark had diminished, indicating the rat's effective self‐recovery from a mild thermal injury at 55 °C.

Subsequently, the synthesized FSH3 hydrogel's healing properties were assessed using a dorsal skin wound model in MRSA‐infected diabetic rats (Figure 6B). The commercially produced 3 m hydrogel and PBS served as positive and negative controls. Wound progress was captured in photographs on days 1, 3, 7, and 14 (Figure 6C). Healing in rats administered with FSH1, FSH3, and FSH3 + NIR exhibited faster healing compared to those treated with PBS and 3 m. Noticeably, after a 14‐day treatment period, a considerable open wound persisted in the 3 m cohort (42.1% wound area), comparable to the PBS cohort (57.2% wound area), highlighting the limited effectiveness of 3 m in healing wounds infected with bacteria. Conversely, the FSH1 and FSH3 groups showed notable wound area reductions to 40.9% and 38.7% at 14 days, demonstrating the hydrogels capacity to expedite wound healing. The group treated with FSH3 + NIR hydrogel demonstrated nearly complete wound closure, with wounds healing almost fully, showing a mean wound area of just 10.4% (Figure S19, Supporting Information).

Additionally, on the 14th day post‐operation, wound tissues were extracted for histological analysis. This process provided insights into the hydrogel's role in wound healing.^[^ ^36^ ^]^ The FSH3 + NIR group demonstrated notably superior healing, with regenerated skin exhibiting fully developed epithelial layers and enhanced formation of new blood vessels, in contrast to both control groups, as shown in Figure 6D.^[^ ^37^ ^]^ Masson staining experiment was subsequently utilized to investigate collagen deposition, an essential component of skin regeneration.^[^ ^38^ ^]^ Dense and well‐arranged collagen fibers, indicative of improved collagen deposition, were observed in the FSH3 + NIR set, as shown in Figure 6E.^[^ ^39^ ^]^ Additionally, CD31, tumor necrosis factor‐alpha (TNF‐α), CD86 and CD206 immunofluorescence staining was performed. Figure 6F reveals significant inflammation in the control cohort, whereas the FSH3 + NIR cohort exhibited reduced TNF‐α and CD86 expression compared to the FSH1 and FSH3 sets, suggesting a diminished inflammatory phase in infected diabetic wounds.^[^ ^40^ ^]^ The decrease in inflammation was also corroborated by myeloperoxidase (MPO) staining of neutrophils, showing minimal inflammation in the FSH3 + NIR set.

Moreover, vascular endothelial growth factor (VEGF) and CD31 methods were used to assess neovascularization.^[^ ^41^ ^]^ As depicted in Figure 6F, the FSH3 + NIR group showed a notably higher rate of new blood vessel growth compared to other sets (Figure S20, Supporting Information). Neovascularization is essential for supplying O_2_ and nutrients to metabolically active wounds, promoting the formation of granulation tissue. Mirroring findings from studies on rat dorsal skin wounds, the FSH3 + NIR cohort exhibited the lowest CD86 expression alongside the highest CD206 expression. A decline in M1 macrophages and an increment in M2 macrophages were observed in the FSH3 + NIR therapy cohort. This resulted in elevated levels of anti‐inflammatory and vasculogenic cellular factors, which facilitated the restoration of MRSA‐infected dorsal dermal wounds.

### 2.9 Healing Efficacy of FSH3 Hydrogel In Vivo on MRSA‐Infected Diabetic Oral Ulcers

**Figure 7:**
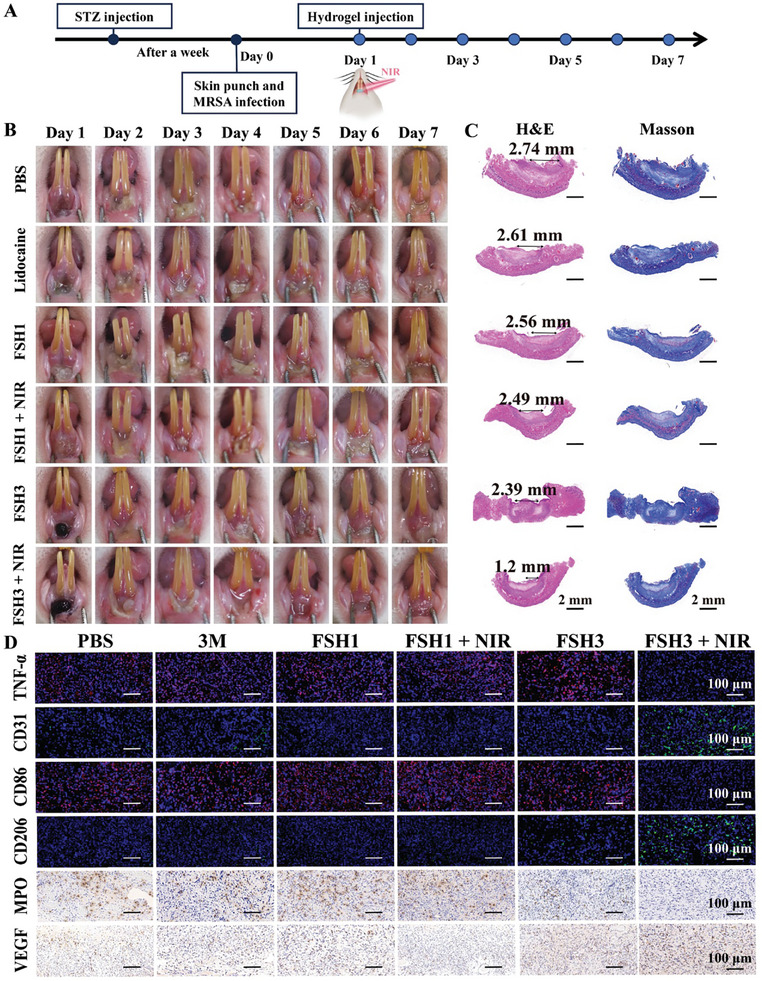
Assessment of the FSH3 hydrogel for treating MRSA‐induced oral mucosa in diabetic rats. A) Procedure for creating the oral ulcer model. B) Digitized images display the process of ulcer healing among the PBS, Lidocaine, FSH1, FSH1 + NIR, FSH3, and FSH3 + NIR groups over a period from day 1 to day 7. C) Tissue sections undergoing H&E and Masson staining. D) Tissue sections showing staining for TNF‐α, CD31, CD86, CD206, MPO, and VEGF at the oral mucosa ulcer location.

Having established the efficacy of the FSH3 hydrogel in healing dorsal skin wounds, the focus shifted to creating an oral ulcer model to further evaluate its healing capabilities. The method for constructing this model is detailed in **Figure** **7**A. An immediate response followed the application of glacial acetic acid to the rat mucosa, marked by a swift whitening at the site. Alongside the hydrogel treatment groups, a PBS‐treated negative control group and Lidocaine serving as a positive control were included. Figure 7B demonstrates that on the first day following the procedure, all rat groups presented with a hard, central ulcer in the mouth, surrounded by swollen, red mucosa and a yellow pseudomembrane. Two days into the hydrogel treatment, a reduction in the pseudomembrane thickness and ulcer size was noted. After four days, the control set still displayed visible remnants of the pseudomembrane, whereas, in the Lidocaine and FSH3 sets, the surrounding mucosal swelling and redness had lessened, with the FSH3 + NIR group showing the smallest ulcer area. By day five, ulcers in the FSH3+ NIR group were almost completely healed.

Following this, after 7 days of therapy, wound specimens were collected from the oral region for morphological and histological examination. Figure 7C shows that in both the PBS and Lidocaine sets, necrosis occurred in the epidermis, accompanied by substantial inflammatory cell infiltration detected. Conversely, the FSH3 group showed reduced presence of inflammatory cells and minimal fibroblast proliferation. Conversely, the FSH3 + NIR group revealed a fully intact epithelium and scant submucosal connective tissue. Masson staining revealed the most pronounced collagen deposition in the FSH3 + NIR set, characterized by dense, orderly fibers.^[^ ^42^ ^]^ The inflammation levels in each group's mucosa were further analyzed through immunohistochemistry (Figure 7D). Maximum TNF‐α positivity was highest in the Lidocaine group, reduced in the FSH3 group, and lowest in the FSH3 + NIR group. This indicates a reduction in the inflammatory reaction observed at the site of the oral ulcer with the FSH3 + NIR treatment.^[^ ^43^ ^]^ Additionally, CD31 staining was applied to identify vascularization within the regenerated oral mucosa. Among all sets, the FSH3 + NIR set showed the most extensive neovascularization and the quickest recovery of wounds (Figure S21, Supporting Information).^[^ ^44^ ^]^

The final stage involved evaluating the biosafety of FSH3, a key aspect of their potential use in wound care. Blood biochemical tests were conducted post‐treatment to assess this. Measurements included red blood cell (RBC), platelet (PLT) counts, white blood cell (WBC), hematocrit (HCT), hemoglobin (HGB), red blood cell distribution width (RDW), mean corpuscular volume (MCV), and mean cell hemoglobin concentration (MCHC) levels. The results indicated no significant variations in these parameters between subjects who were treated with FSH3 and those in the blank control set, suggesting the FSH3 hydrogel's compatibility with blood, as seen in Figure S22 (Supporting Information). Additionally, hematoxylin‐eosin (H&E) staining was used to investigate the potential organ toxicity of the FSH3 hydrogel dressing in rats. Organ tissues from rats treated with the hydrogel showed no substantial inflammatory cell presence compared to healthy, untreated tissues (Figure S23, Supporting Information). These results indicate that the FSH3 hydrogel displays negligible biological toxicity toward healthy in vivo tissues. Collectively, such observations highlight the positive biosafety profile of the FSH3, positioning it as a viable option for clinical application in oral wound healing.

### 2.10 Transcriptomic Evaluation of Antibacterial Mechanism of FSH3 Hydrogel

**Figure 8:**
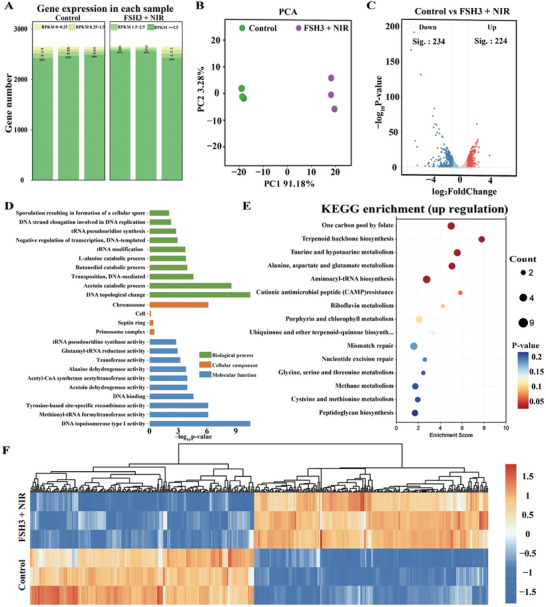
RNA sequencing analysis for NIR‐enhanced FSH3 treatment: A) Quantitative evaluation of DEGs from the data. B) PCA analysis displaying DEGs detected by RNA‐seq between the control and FSH3 + NIR sets. C) Volcano plots displaying DEGs (gray indicates non‐significant genes, red signifies upregulated genes, blue denotes downregulated genes). D) GO enrichment profiling of upregulated genes. E) KEGG pathway profiling for genes that are upregulated. F) Heatmap visualization of the DEGs.

Metagenomics utilizes genomic methodologies and bioinformatics tools to explore the genetic content of entire microbial communities.^[^ ^45^ ^]^ The antibacterial effects of FSH3 + NIR were analyzed through RNA sequencing, which identified and studied the differentially expressed genes (DEGs) in MRSA following exposure to FSH3 + NIR. The distribution of gene expression counts and values in the samples is depicted in **Figure** **8**A. The principal component analysis confirmed the internal consistency of group variances, thereby validating the RNA sequencing findings (Figure 8B).

In comparison to the control group, the FSH3 + NIR group exhibited a total of 458 differentially expressed genes, with 234 genes being down‐regulated and 224 genes being up‐regulated (Figure 8C). Gene ontology (GO) analyses revealed that these DEGs were linked to bioprocesses, molecular functionalities and cellular components (Figure 8D). Furthermore, the Kyoto Encyclopedia of Genes and Genomes (KEGG) analysis, displayed in Figure 8E, shows these DEGs participating in 30 distinct KEGG pathways. Notably, DEGs in the FSH3 + NIR and control groups were particularly abundant in pathways related to Translation, Metabolism of cofactors and vitamins, and Replication and repair, including pathways like one‐carbon pool by folate, aminoacyl‐tRNA biosynthesis, and mismatch repair. These pathways are associated with the synthesis of bacterial nucleic acids.^[^ ^33a^ ^]^ Additionally, the DEGs were linked to amino acid synthesis, crucial for protein synthesis, metabolic regulation, and the structural and functional integrity of MRSA. The transcriptomic disparity between the FSH3 + NIR treated and control groups was significant, as shown in Figure 8F. Overall, the changes suggest that the FSH3 + NIR treatment impacts the growth and reproduction of MRSA by modifying pathways related to Genetic Information Processing and Metabolism.

## 3 Conclusion

Our study presents the FSH3 hydrogel patch method for creating a robust adhesive patch designed for treating oral ulcers. FSH3 consists of methacrylate SF, NB‐modified hyaluronic acid, and ferric iron/shikonin nanoparticles, which enhance both adhesion duration and healing effectiveness. By a simple local injection, a layer of adhesive hydrogel covers mucosal imperfections upon exposure to 365 nm UV light. The FSH3 then continuously releases bioactive compounds that serve antibacterial, anti‐inflammatory, and antioxidative functions, markedly improving the management of oral mucosa defects. Moreover, the FSH3 is fully bioabsorbable and displays minimal toxicity once it has served its therapeutic function. The efficacy of FSH3 in promoting wound healing in both back and oral sites was shown through a type 1 diabetic rat model. We contend that the FSH3 framework outlined in our study may enhance the creation of hydrogel adhesives and inspire innovative designs for wounds that utilize bioactive materials derived from nature.

## 4 Experimental Section

### Materials

Hyaluronic acid (HA, purity > 99%, and Mw = 500 kDa), lithium phenyl(2,4,6‐trimethylbenzoyl)phosphinate (LAP), 4‐(4,6‐dimethoxy‐1,3,5‐triazin‐2‐yl)‐4‐methyl morpholinium chloride (DTMPC), lithium bromide (LiBr), sodium carbonate, glycidyl methacrylate, ethanol and N, N‐dimethylformamide were provided by Aladdin (Shanghai, China). Silkworm cocoons were provided by the Chongqing Sericulture Technology Promotion Center (China). Shikonin (SK) was purchased from PureChem (Chengdu, Sichuan). N‐(2‐aminoethyl)‐4‐(4‐(hydroxymethyl)‐2‐methoxy‐5‐nitrophenoxy)butanamide (NB‐NH_2_) was provided by Chaocheng (Jiaxing, China). Ferric nitrate [Fe(NO_3_)_3_], glutaraldehyde and ethyl acetate were purchased from Macklin (Hangzhou, China). Innochem (Beijing, China) supplied propidium iodide (PI). Dimethyl sulfoxide (DMSO), 1,1‐diphenyl‐2‐picrylhydrazyl (DPPH) was sourced from Innochem (Shanghai, China). Thermo Fisher (L7012, Waltham, USA) provided a bacterial viability kit containing PI and SYTO9. Beyotime (Shanghai, China) provided various items, including 2′,7′‐dichlorofluorescein diacetate, fetal bovine serum, Dulbecco's modified Eagle's medium (DMEM), 2,2ʹ‐azino‐bis(3‐ethylbenzothiazoline‐6‐sulfonic acid) diammonium salt (ABTS), and phosphate‐buffered saline (PBS, pH = 7.4). Media and supplements such as CCK‐8, penicillin‐streptomycin, and trypsin‐EDTA were supplied by Gibco (Wenzhou, China). Hopebiol (Shanghai, China) provided Tryptone Soya Broth and Triton X‐100. Agar powder, streptozotocin (STZ), and Luria‐Bertani Broth came from Solarbio (Beijing, China). Yeasen (Wenzhou, China) supplied calcein acetoxymethyl ester (calcein‐AM). Unless specified otherwise, all solvents and reagents of analytical grade were utilized as supplied.

### Fabrication of Fe^3+^/SK (FeSK) Nanoparticles

Initially, 10 mg of SK was dissolved in a 5 mL N, N‐dimethylformamide solution. Subsequently, 1 mL of a 5 mg Fe(NO_3_)_3_ solution was introduced, followed by 25 mL of ethyl acetate. The mixture was agitated for a period of 8 h at a temperature corresponding to the ambient environment. Next, a centrifuge process at 9800 rpm for 6 min served as the concluding step. The particles obtained were thrice rinsed with ethanol and then resuspended in double distilled water (DDW) to form the final product.

### Synthesis of Nitrobenzyl‐Modified Hyaluronic Acid and Methacrylate Silk Fibroin

Synthesis of nitrobenzyl‐modified hyaluronic acid (HA‐NB): Hyaluronic acid, 2 g, was dissolved in 100 mL of 0.01 m 2‐morpholinoethanesulfonic acid solution, maintaining a pH of 5.17. Then, 0.4 g of DTMPC was added to the solution. Following that, 60 mg of NB‐NH_2_ was mixed into DMSO before being combined with the earlier mixture. This combined mixture was stirred for 14 h at 30 °C in a dark setting. The subsequent process involved dialyzing the mixture against DDW for 3 days, followed by freezing and lyophilization.

### Synthesis of Methacrylate Silk Fibroin

Silk fibroin (SF) extraction began with cutting silkworm cocoons into strips and boiling them in sodium carbonate solution (0.02 m) for 1 h to remove the sericin layer. The degummed fibers were then washed with DDW, air‐dried at 37 °C, and soluble in LiBr (9.3 m) at 60 °C for 5 h. The SF solution was then placed in a Solarbio dialysis bag (3500 Da, Shanghai, China) and dialyzed against DDW for 48 h to remove LiBr. Following dialysis, the solution was centrifuged at 9500 rpm for 25 min at 4 °C, a procedure that was replicated twice. The final step in the preparation was freeze‐drying to obtain SF. To create methacrylate SF (SFMA), lyophilized SF (10 g) was mixed with PBS (50 mL) and agitated for 3 h. Concurrently, glycidyl methacrylate (2.93 g) was combined with pH 8.5 PBS buffer (120 mL) and agitated continuously in a separate container. The reaction between glycidyl methacrylate and SF was permitted to continue at room temperature throughout the night, after which the solution underwent dialysis in bags with a molecular weight cut‐off of 3500 Da in deionized water for 6 days. The SFMA obtained post‐dialysis was lyophilized for future applications.

### Preparation of FSH Hydrogels

In this study, hybrid hydrogels composed of FeSK nanoparticles, SFMA, and HA‐NB (termed FSH) were synthesized. Initially, 1 mL of the SFMA solution (10%, w/v), which included 8 mg of the LAP, was blended with 1 mL of HA‐NB solution (1%, w/v) containing different concentrations of FeSK nanoparticles. Upon exposure to UV light for 20 s, the mixture underwent a double‐bond polymerization, leading to the formation of FSH hydrogel. In total, four distinct FSH hydrogels were prepared, with varying concentrations of FeSK: 0, 0.5, 1, and 2 mg mL^−1^; they were designated as FSH1, FSH2, FSH3, and FSH4.

### Physicochemical Properties Characterization

The objective of this analysis was to ascertain the presence of functional groups in the samples, for which a Fourier transform infrared (FTIR) spectrometer from Bruker (Tensor II, Germany) was used to evaluate the functional groups in the samples, examining the spectral region between 400 and 4000 cm^−1^. Deuterium oxide solvent was employed to prepare samples for nuclear magnetic resonance (NMR) experiment, subsequently processed with a Quantum‐I instrument (400 MHz, China). ^1^H NMR spectra data were acquired and evaluated through Mestrenova. In the quantitative analysis of ^1^H NMR spectroscopy, where dimethyl terephthalate functioned as the internal benchmark, a relaxation delay time of 10 s was established.

Thermogravimetric analysis (TGA) was performed over a temperature range from 30 to 600 °C, using a heating rate of 15 °C per minute. The point of degradation was identified when a 2 wt.% weight loss occurred, using a sample weighing 5 mg. These assessments were carried out using a TGA8000 instrument from PerkinElmer (USA). For the measurements, specimens were placed in a platinum pan, with an airflow maintained at 20 mL mi^−1^n.

Assessments of zeta potentials and dynamic light scattering (DLS) were conducted employing a Malvern device (UK). Analysis of autocorrelation functions, using the cumulants method, provided the average particle diameters. For every sample, at least three assessments were carried out at 37 °C, preceded by a 180‐s stabilization period before commencing each measurement.

Measurements of ultraviolet–visible–near‐infrared (UV–vis–NIR) spectra were performed employing a Cary5000 spectrophotometer by Agilent (USA) at ambient temperature.

Adhesion tests for FSH hydrogels on biological tissues utilized fresh pig skin, adhering to ASTM standards. Pork skin slices underwent preparation and an overnight EDC/NHS treatment. Subsequently, a 200 µL volume of SFMA/HA‐NB precursor was dispensed onto a 10 mm × 10 mm area on two pieces of pig skin, followed by cross‐linking using 365 nm light for 50 s. The tensile strength of the formed FSH hydrogels was then evaluated up to the point of detachment. These evaluations employed an Instron 5944 apparatus, executing a stretch rate of 8 mm min^−1^.

For scanning electron microscope (SEM) imaging, specimens were processed employing a Hitachi device (SU8010, Japan), with the voltage set to an accelerating rate of 5 kV. These samples were initially shock‐frozen in liquid nitrogen and subsequently transferred to a freeze dryer for lyophilization over a period of 24 h. Once lyophilized, the specimens were fractured delicately in liquid nitrogen and mounted on aluminum stubs. Prior to SEM analysis, the surface of each sample was coated with a 6 nm layer of platinum.

To assess the swelling characteristics, freeze‐dried FSH hydrogel specimens that were pre‐weighed (M_o_) were submerged in PBS at 37 °C until they reached equilibrium. At designated intervals during swelling, FSH hydrogels were removed, surface moisture was gently blotted off with wet filter tissues, and their wet weights (M_p_) were noted employing a Sartorius BSA224S‐CW balance (Germany). The swelling ratio was then determined using the formula (M_p_ – M_o_)/M_o_, where M_p_ refers to the weight of the completely swelled sample and M_o_ to the weight after lyophilization.

For assessing water retention capacity, freeze‐dried FSH hydrogel samples were submerged in PBS until water uptake equilibrium, labeled as M_z_, was reached. Next, these samples at equilibrium were transferred to a 37 ·C oven, and their weights (M_x_) were recorded at specified intervals. The percentage of water retention in the FSH hydrogels was calculated using the following formula: Water Retention (%) = (1 – M_x_/M_z_) × 100%.

To assess the degradation characteristics of the crafted FSH hydrogels, the specimens were subjected to an initial immersion in PBS until they reached water uptake equilibrium. At specific time intervals, the morphology and mass variations of the FSH hydrogels cultured in PBS were captured via photographs and measured for weight. The degradation percentage was determined using the formula: Degradation Ratio (%) = (1– M_h_/M_j_) × 100%, wherein M_h_ denotes the weight of the FSH at a certain time, and M_j_ represents its weight when fully swollen.

The photothermal efficiency of the produced nanoparticles and hydrogels was tested employing an 808 nm NIR laser. Each sample was positioned in 2.5 mL tubes and subjected to an 808 nm laser for specified durations at different NIR power levels (0.5, 1.0, and 2.0 W cm^−2^). A FLIR E4 infrared thermometer (USA) recorded the temperature changes during these irradiation sessions. Additionally, the photothermal stability of the samples was examined over four cycles of heating and cooling.

Following the acquisition of the heating and cooling curves of FeSK (1 mg mL^−1^) under 808 nm laser irradiation (1 W cm^−2^), the photothermal efficiency (η) was calculated in accordance with the following equation.

(1) $$\eta =\frac{hS\left(T_{\max}-T_{\mathrm{surr}}\right)-Q_{0}}{I\left(1-10^{-A808}\right)}$$

(2) $$\tau_{s}=\frac{m_{d}C_{d}}{hS}$$

(3) $$Q_{0}=hS\left(T_{\max,\mathrm{water}}-T_{\mathrm{surr}}\right)$$

The thermal time constant, τ_s_, can be calculated from the linear regression curve of the cooling curve. The mass, m_d_, and heat capacity of the solution, C_d_, are represented by the respective symbols. The value of *hS* can be calculated according to Equation 2. The steady‐state maximum temperature of the water (*T*_max, water_) and the ambient room temperature (*T*_surr_) can be calculated according to Equation 3, as can Q_0_. I and A808 represent the laser power and sample absorbance at 808 nm, respectively. The photothermal efficiency (η) can be calculated according to the equations:

A DHR‐2 stress‐controlled rheometer (manufactured by TA Instruments, USA) was used for the analysis of the rheological characteristics of FSH hydrogels at ambient temperature. The hydrogel precursor was injected into a 25 mm diameter, 1 mm height circular plastic mold to create samples fitting the rheometer's shear disc. Initially, a strain sweep test was performed on the cured samples at 25 °C, incrementally increasing the oscillatory strain from 0.1% to 1000% to identify the linear viscoelastic region. Next, frequency sweep trials were done at 25 °C to explore how frequency (ranging from 0.1 to 10 Hz) affected the modulus, including both the storage modulus (G′) and loss modulus (G″). The formation time of the hydrogel was then determined through a dynamic oscillation time sweep at a fixed strain (1%) and frequency (0.1 Hz),^[^ ^17^ ^]^ and was subsequently assessed through a series of dynamic stepped tests conducted at varying oscillatory strains (1–400%) to evaluate the hydrogel's self‐healing ability. Lastly, experiments were conducted to examine the viscous behavior of the hydrogel in shear thinning, with shear rates that ranged from 0.1 1/s to 10 1/s.

### ROS Scavenging Ability

Moreover, the FSH samples radical scavenging potential was assessed via the DPPH assay. DPPH was dissolved in an 80:20 ethanol‐water solution at 200 µm. Specimens were submerged in the DPPH solution and incubated at 37 °C in darkness. Following a designated period, the absorbance of the reaction mixture at 517 nm was quantified, calculating the DPPH scavenging ability as Inhibition (%) = (1 − A_b_/A_v_) × 100%, where A_v_ represents the starting DPPH absorbance, and A_b_ denotes the absorbance following incubation with the samples. Besides, the total antioxidant feature of the FSH hydrogels was also evaluated by adopting the ABTS assay. Hydrogels (50 mg) with different nanoparticle concentrations were soaked in a 1 mL culture medium for 12 h. Their extracts were then analyzed using a total antioxidant capacity assay kit, and absorbance at 414 nm was measured.

### In Vitro Antibacterial Assays

The antibacterial effectiveness of FSH hydrogels in vitro against MRSA and MRPA was evaluated using the spread plate technique. Initially, 8 mm diameter hydrogel pieces were saturated in PBS to achieve absorption equilibrium, followed by sterilization under a UV lamp for 30 min. These pieces were then submerged in a 1 mL bacteria suspension (1.0 × 10^8^ CFU mL^−1^). After 4 h of incubation at 37 °C, some samples underwent NIR irradiation (808 nm, 1 Wcm^2^) for 3 min. Subsequently, 100 µL of the bacterial mix was extracted from each set and diluted, and 100 µL of this dilution was evenly dispersed on fresh agar plates. The plates were cultured overnight, and the bacterial colonies were recorded and enumerated using a J3 colony counter (Tenlin, China). For comparison, a control group consisting of the bacteria suspension was treated similarly with PBS, with or without NIR exposure.

Besides, the bactericidal capabilities of the FSH specimens were estimated by bacterial live/dead staining. Subsequent to the implementation of distinct methodologies, bacterial cells were subjected to a 20‐min incubation period with PI and SYTO9 for 20 min in the absence of light, followed by three washes with PBS.^[^ ^18^ ^]^ In accordance with the instructions outlined by the manufacturer, SYTO9 demonstrated efficacy in labeling all bacteria exhibiting green fluorescent properties. Conversely, PI exhibited preferential staining of dead bacteria, displaying red fluorescence. Fluorescence microscopy images were then acquired using a Nikon A1 confocal laser scanning microscopy system (Japan).

Furthermore, SEM imaging techniques were utilized to observe the morphological alterations in bacteria following antibacterial tests. In the SEM procedure, bacterial suspensions of MRPA and MRSA were gathered and rinsed thrice with PBS. The bacteria were then preserved at 4 °C in 2.5% glutaraldehyde overnight, followed by dehydration through graded ethanol concentrations for SEM examination.

Additionally, the impact of FSH hydrogels on MRSA biofilms underwent assessment. The introduction of FSH hydrogels to MRSA biofilms, which were cultivated in 24‐well plates, followed a specific treatment protocol.^[^ ^19^ ^]^ Subsequently, the growth medium was discarded, and the biofilms underwent triple washing with PBS. Methanol (1 mL) served to fix the MRSA biofilm for a quarter‐hour. Following methanol's removal, the resultant sediment dried naturally. After this, a staining process using 0.1% crystal violet solution (1 mL) ensued for 25 min. To eliminate surplus dye, the biofilm faced three additional PBS washes. The petri dishes, turned upside down on filter paper, allowed for moisture removal. Once the Petri plates dried, a solution of 33% acetic acid (1 mL) was applied to dissolve the crystal violet, maintained at 37 °C for half an hour, before measuring absorbance at 590 nm.

### Experiments on Cell Survival

The cytotoxic effects of the developed FSH hydrogels were evaluated through the utilization of CCK‐8 assays. Before the commencement of the tests, the hydrogels were subjected to UV sterilization and subsequently immersed in a DMEM medium containing 10% fetal bovine serum and 1% penicillin‐streptomycin for 1, 3, and 5 days to produce leachates. Concurrently, RS1 cells were plated in a 96‐well plate at a density of 6 × 10^3^ cells per well. The existing culture medium in each well was removed after an overnight incubation period. Subsequently, 100 µL of leachate was added to each well, and the cells underwent incubation for 12 h. Afterward, a fresh solution containing CCK‐8 replaced the leachate, and the cells were subsequently incubated for an additional 2 h. Absorbance at 450 nm was then detected by employing a Thermo Fisher 1530 microplate reader from Finland. Additionally, the viability of RS1 cells exposed to the FSH hydrogels was evaluated by employing a calcein‐AM/PI cell viability probe, according to the manufacturer's guidelines.^[^ ^20^ ^]^ Cells were prepared and seeded in the manner previously outlined. Post‐treatment, cells from different groups were stained with PI and calcein‐AM for 20 min. Fluorescent microscopy was used to image the stained cells. Dead cells emitted red fluorescence due to compromised plasma membranes, while living cells showed green fluorescence indicative of active esterase.

### Evaluation of Antioxidant Effects

The materials capacity to shield against oxidative stress within the cell was determined by observing cell viability under H_2_O_2_ exposure after treatment with FeSK nanoparticles and FSH hydrogels. RS1, RAW 264.7 and HGF cells were seeded at 2 × 10^4^ cells per well in 96‐well plates and permitted to grow overnight. Cells exposed to H_2_O_2_ in the medium acted as the positive control set. Different treatments were then applied to the H_2_O_2_‐containing culture medium, with a subsequent incubation period of 6 h. Cell viability was subsequently gauged employing the CCK‐8 experiment. 2′,7′‐dichlorofluorescein diacetate (DCFH‐DA) was used to assess intracellular ROS levels. Round culture dishes were used to culture RS1, RAW 264.7 and HGF cells overnight. Post‐medium removal, cells received identical volumes of either hydrogel extract or H_2_O_2_ for 6 h. DCFH‐DA was incorporated following the manufacturer's guidelines. Subsequently, cells were visualized using a confocal laser scanning microscope from Leica (STELLARIS 5, Germany).

### Assessment of Cell Migration

The impact of FSH hydrogel on cell migration characteristics was evaluated through a scratch experiment, with RS1 cells dispersed in 6‐well plates at 2 × 10^5^ cells per well. Upon achieving 90% confluence, a vertical linear scratch was created, adopting a 100 µL pipette tip to generate artificial gaps. Subsequent to this, detached cells were eliminated by rinsing the cells four times with PBS. Fresh medium containing equal amounts of H_2_O_2_ or FSH extract was then applied to the RS1 cells. Following incubation periods of 0, 12, and 24 h, the cells underwent a 30‐min fixation process using paraformaldehyde. Finally, cell migration was monitored through microscopic examination.

### Evaluation of Angiogenesis

The angiogenesis assays were conducted to assess cell‐induced blood vessel formation under oxidative stress conditions, both before and after applying the materials. At first, 10 µL of Corning 356234 Matrigel (China) was uniformly dispersed over every well of a 96‐well plate and permitted to solidify for 90 min at 37 °C. HUVEC (human umbilical vein endothelial cells) were subsequently seeded onto the Matrigel (Corning, cell density = 1 × 10^4^ cells per well). Once cell attachment to the well surface was confirmed, either H_2_O_2_ or H_2_O_2_ soaked in FSH hydrogel was incorporated. Cells underwent cultivation for durations of 8 h, after which microscopic analysis was conducted to record the formation of new vascular structures.

### In Vitro Blood Compatibility Assay

The hemolytic potential of newly formulated FSH specimens was assessed with rat red blood cells (RBCs). These cells were obtained by centrifuging rat fresh blood with ascorbic acid (2%) for 12 min, followed by three washes with PBS. A volume of 1 mL of the diluted RBCs was then placed into a test tube. Afterward, 100 mg of the hydrogel samples were added to these tubes. Additionally, for control purposes, positive and negative controls were utilized in the experiment, comprising 100 mg of 0.1% Triton X‐100 and PBS, respectively. Following a 3‐h incubation at 37 °C, the suspensions underwent centrifugation at 2600 rpm for 5 min. The absorbance of the supernatants was subsequently measured at 545 nm. The hemolysis ratio was calculated using the equation: Hemolysis Ratio (%) = [(*A*_3_ − *A*_2_)/(*A*_1_ − *A*_2_)] × 100%, where *A*_1_ means the absorbance for the Triton‐treated specimen, *A*_2_ for the PBS‐treated sample, and *A*_3_ for the hydrogel‐treated sample.

### Development of Type 1 Diabetic Rats

The animal study complied with the guidelines and standards established by the Institutional Animal Care and Use Committee of Wenzhou Medical University and followed the National Research Council's Guide for the Care and Use of Laboratory Animals during its implementation.

Male Sprague–Dawley rats supplied by Beijing Vital River (China) were employed for the investigation. Rats weighing ≈255 g and having fasted for 12 h were administered a 1% STZ intraperitoneal injection. One week post‐injection, diabetes induction was verified by measuring the blood glucose level from tail vein samples. The induction of diabetes was confirmed in rats exhibiting blood glucose levels exceeding 16.7 mM.

### Development of MRSA‐Infected Dorsal Skin Wounds

Initially, the efficacy of FSH hydrogels was evaluated in vivo on the dorsal skin of diabetic rats. To establish a bacterial‐infected wound model, four circular, full‐thickness wounds (8 mm in diameter) were induced on the dorsal area of each rat. Then, 10 µL of MRSA culture (1.0 × 10^7^, CFU mL^−1^) was applied to the wounds and spread evenly. Various treatments were applied to these wounds on the following day: 100 mg of PBS, 3 m, FSH1, FSH3, FSH1 with NIR (1 W cm^−2^, 3 min), and FSH3 hydrogel with NIR (1 W cm^−2^, 3 min). Hydrogels were secured in place using medical tape. Defined intervals captured digital images of wounds (days 1, 3, 7, and 14) to monitor the process of wound healing.

### Development of MRSA‐Infected Oral Ulcer Wounds

Further evaluation of FSH hydrogels' efficacy was conducted on the oral ulcers of diabetic rats. Briefly, the mandibular side mucosa of male Sprague–Dawley diabetic rats was initially dried using sterile cotton balls. Oral ulcers were then induced by applying 3 mm by 3 mm filter papers soaked in 40% acetic acid to the oral mucosa for 30 s. After removing the filter paper, a sterile cotton ball soaked in physiological saline was used to dab the area, removing any residual acetic acid. Two days post‐ulcer induction (day 1), sites with evident mucosal defects were treated with a 5 µL bacterial solution (1 × 10^7^, CFU mL^−1^) and segregated into three treatment groups. Each group received an application of 45 mg of FSH hydrogels to the oral ulcers. The study included six groups: a blank control with PBS, a positive control with Lidocaine hydrogel, an FSH1 hydrogel set, and an FSH1 hydrogel set subjected to laser exposure (1.0 W cm^−2^, 3 min), an FSH3 hydrogel set, and an FSH3 hydrogel set subjected to laser exposure (1.0 W cm^−2^, 3 min). Daily observations were made post‐treatment, and the rats were euthanized on day 8 for additional investigations. To ensure unbiased results, different researchers conducted the surgeries and subsequent evaluations.

### Assessment of Hemostatic Effectiveness

In the beginning, the hemostatic properties of the constructed FSH specimens were evaluated by adopting a rat tail bleeding model. The lower third of the rat's tail was excised with surgical scissors. After 15 s, 200 mg of distinct specimens were introduced into the resulting wound, with the subsequent blood loss documented photographically. For further examination of the hemostatic capabilities of FSH specimens, a liver hemorrhage model was created. Typically, an incision was made in the abdomen of a rat to expose the liver, and any released fluid was meticulously blotted with absorbent paper. Following this, a scalpel was employed to make a 2 mm deep incision that extended 10 mm along the liver's surface. Subsequently, 200 mg of different test materials were applied to the liver wound, and relevant photographs were taken while the extent of blood loss was quantified.

### Testing Photothermal Activity

Groups of diabetic rats were assigned randomly into six categories, with each group containing three rats: PBS, 3 M, FSH1, FSH3, FSH1 + NIR, and FSH3 + NIR. Wounds treated with FSH1 and FSH3 hydrogels underwent NIR exposure at 1 W cm^−2^ for 3 min. Thermal images of these regions were acquired by adopting a thermal infrared camera to assess the photothermal response.^[^ ^21^ ^]^

### Assessment of Degradation

The degradation characteristics of the fabricated hydrogel were studied by adopting a rat subcutaneous implantation model. A sterilized FSH3 hydrogel sheet (8 mm in diameter) was implanted under the skin on the rat's back. Changes in morphology and weight of the implanted hydrogel were documented and measured at specific intervals. The degradation percentage was computed with the formula: Degradation Ratio (%) = (1 – *W*_d1_/*W*_d2_) × 100%, where *W*_d1_ denotes the weight of the hydrogel at a specific time, while *W*_d2_ indicates the weight of the fully swollen hydrogel.

### Evaluation of Biosafety

After conducting in vivo experiments on bacteria‐infected wounds, blood samples were collected from rats for both blood biochemistry analyses and standard blood tests. At the same time, critical organs, including the spleen, kidney, lung, heart, and liver, were extracted from the rats and characterized histologically using H&E staining.^[^ ^22^ ^]^ Additionally, blood biochemical parameters were assessed using ocular vein blood samples collected after various treatments.

### Analysis of Histology

After concluding the experiment, wounds from both the oral mucosa and back skin were excised for histological analysis. The tissues were sliced into 40 µm sections using a Leica microtome (Germany). Then, these samples were immersed in 4% paraformaldehyde for 30 h and subsequently encased in paraffin. Following preparation, the tissue sections were stained with CD31, H&E, Masson's trichrome, MPO, TNF‐α, VEGF, CD206 (M2), and CD86 (M1).

### Statistical Analysis

Three unbiased investigators, unaware of group allocations, conducted analyses on both macroscopic and histological outcomes. Experimental data were represented as mean ± standard deviation (SD). The statistical assessment utilized Student's t‐test for analysis, deeming differences significant at *P < 0.05, **P < 0.01, or ***P < 0.001. A notation of NS indicated P > 0.05.

## Conflict of Interest

The authors declare no conflict of interest.

## Supporting information



Supporting Information

## Data Availability

The data that support the findings of this study are available from the corresponding author upon reasonable request.
