# Supplementary material for: Ferric Iron/Shikonin Nanoparticle‐Embedded Hydrogels with Robust Adhesion and Healing Functions for Treating Oral Ulcers in Diabetes
Source: Adv Sci (Weinh). 2024 Oct 11;11(45):2405463. doi: 10.1002/advs.202405463 (PMC11615794; doi:10.1002/advs.202405463)
Supplement: Supplementary file 1 — Supporting Information [file ADVS-11-2405463-s001.docx]

**Supplementary Material**

**Ferric Iron/Shikonin Nanoparticle-Embedded Hydrogels with Robust Adhesion and Healing Functions for Treating Oral Ulcers in Diabetes**

Xiaojing Chen^1#^, Zhangping Li^2#^, XinXin Ge^3^, Xiaoliang Qi^4*^, Yajing Xiang^3^, Yizuo Shi^3^, Ying Li^5^, Yao Pan^1^, Yingying Wang^1^, Yiyu Ru^1^, Kelei Huang^1^, Jiatan Shao^1^, Jianliang Shen^4,5*^, He Li^1*^

^1^Department of Otolaryngology, The First Affiliated Hospital of Wenzhou Medical University, Wenzhou, Zhejiang 325000, China

^2^The Quzhou Affiliated Hospital of Wenzhou Medical University, Quzhou People’s Hospital, Quzhou 324000, China

^3^School & Hospital of Stomatology, Wenzhou Medical University, Wenzhou, Zhejiang 325027, China

^4^National Engineering Research Center of Ophthalmology and Optometry, Eye Hospital, Wenzhou Medical University, Wenzhou, Zhejiang 325027, China

^5^Zhejiang Engineering Research Center for Tissue Repair Materials, Wenzhou Institute, University of Chinese Academy of Sciences, Wenzhou, Zhejiang 325001, China

AUTHOR INFORMATION

All authors have approved the final version of this manuscript.

^#^These authors contributed equally to this work.

^*^To whom correspondence should be addressed: xiaoliangqi90@gmail.com (X. Qi), shenjl@wiucas.ac.cn (J. Shen), lihe@wzhospital.cn (H. Li).


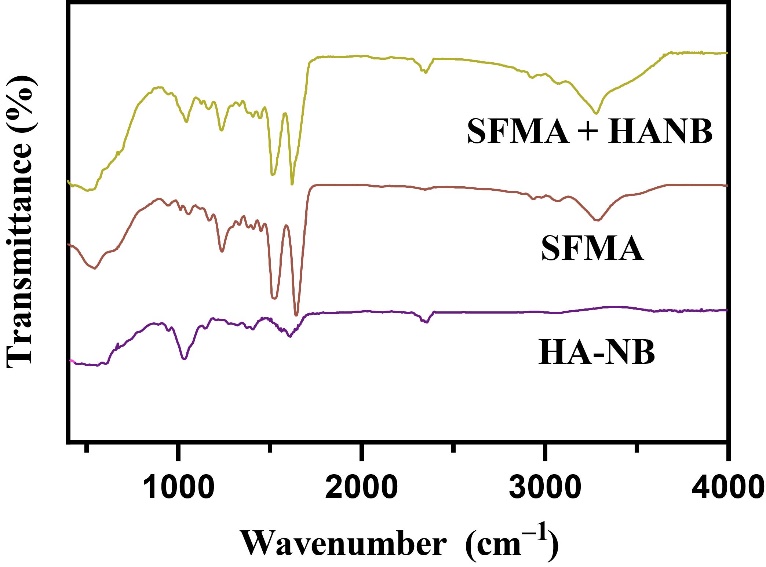
**1. Supplementary Figures**

**Figure S1.** Analyzing HA-NB and SFMA hydrogels using FTIR spectroscopy data.


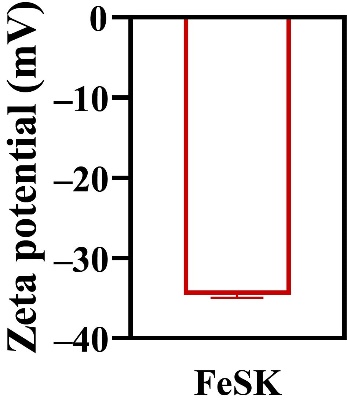


**Figure S2.** Zeta potential measurements for FeSK nanoparticles, shown with error bars indicating mean ± SD (n = 3).


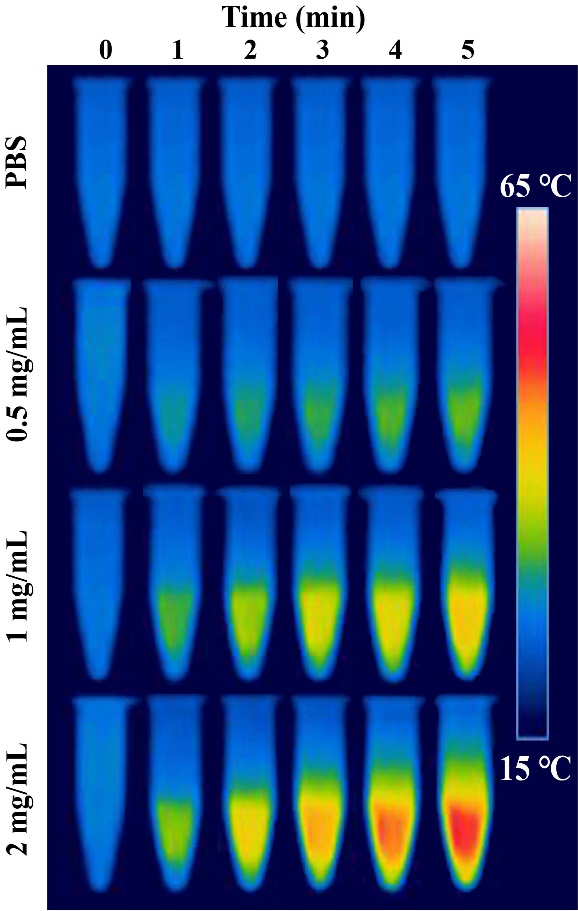
**Figure S3.** Photothermal response of FSH hydrogels under 808 nm NIR exposure (1 W/cm^2^ for 10 minutes) depicted in images.


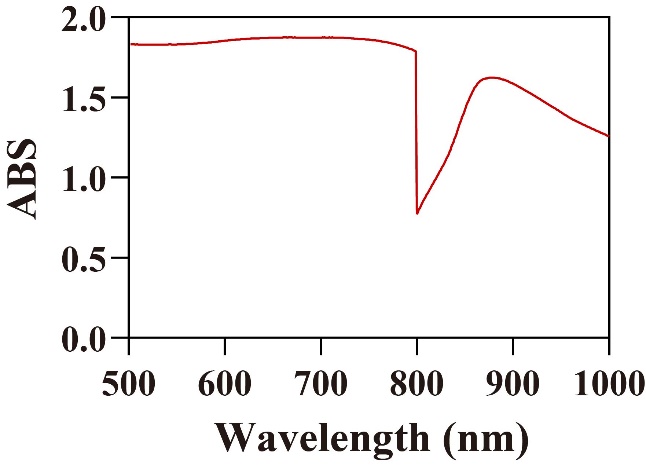
**Figure S4.** UV–VIS–NIR spectral analysis of FeSK nanoparticles at a concentration of 1 mg/mL.


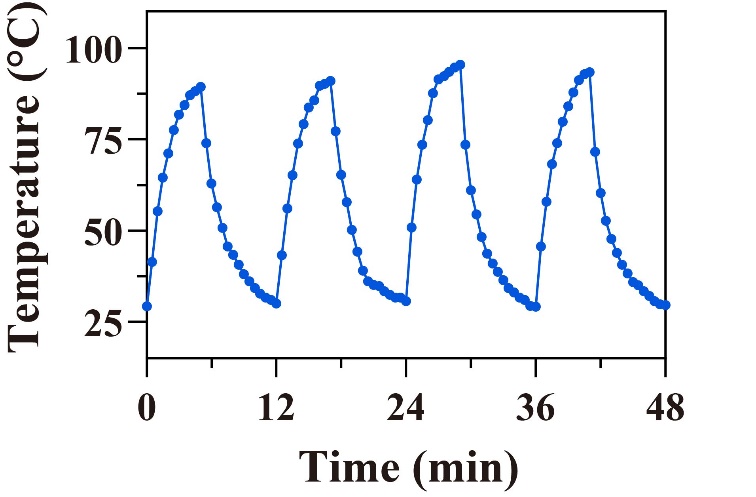
**Figure S5.** Curve depicting photothermal cycling of FeSK.


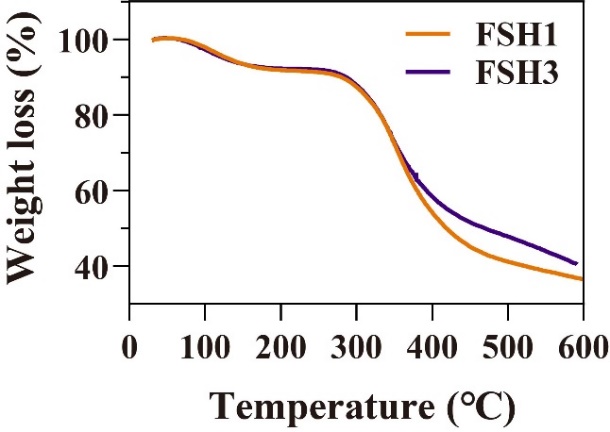
**Figure S6.** TGA profiles for FSH hydrogels.


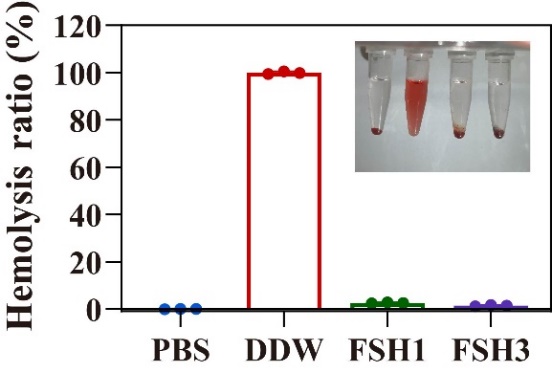


**Figure S7.** Evaluation of hemolysis and photographs of FSH hydrogels following a 2-hour exposure to erythrocytes at 37 °C, displayed with mean ± SD error bars (n = 3).


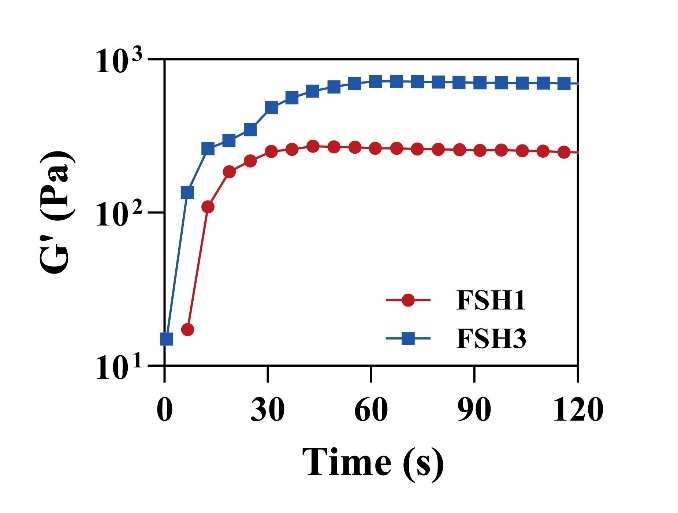
**Figure S8.** Oscillatory time sweep tests of FSH hydrogels.


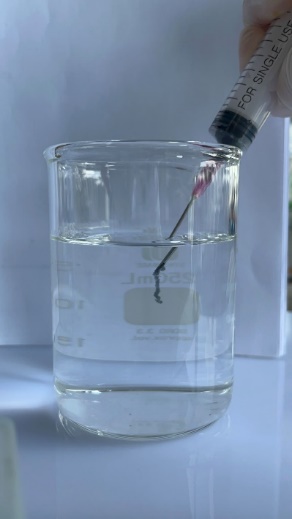
**Figure S9.** Depiction of the injectability of FSH3 hydrogel in a PBS environment.


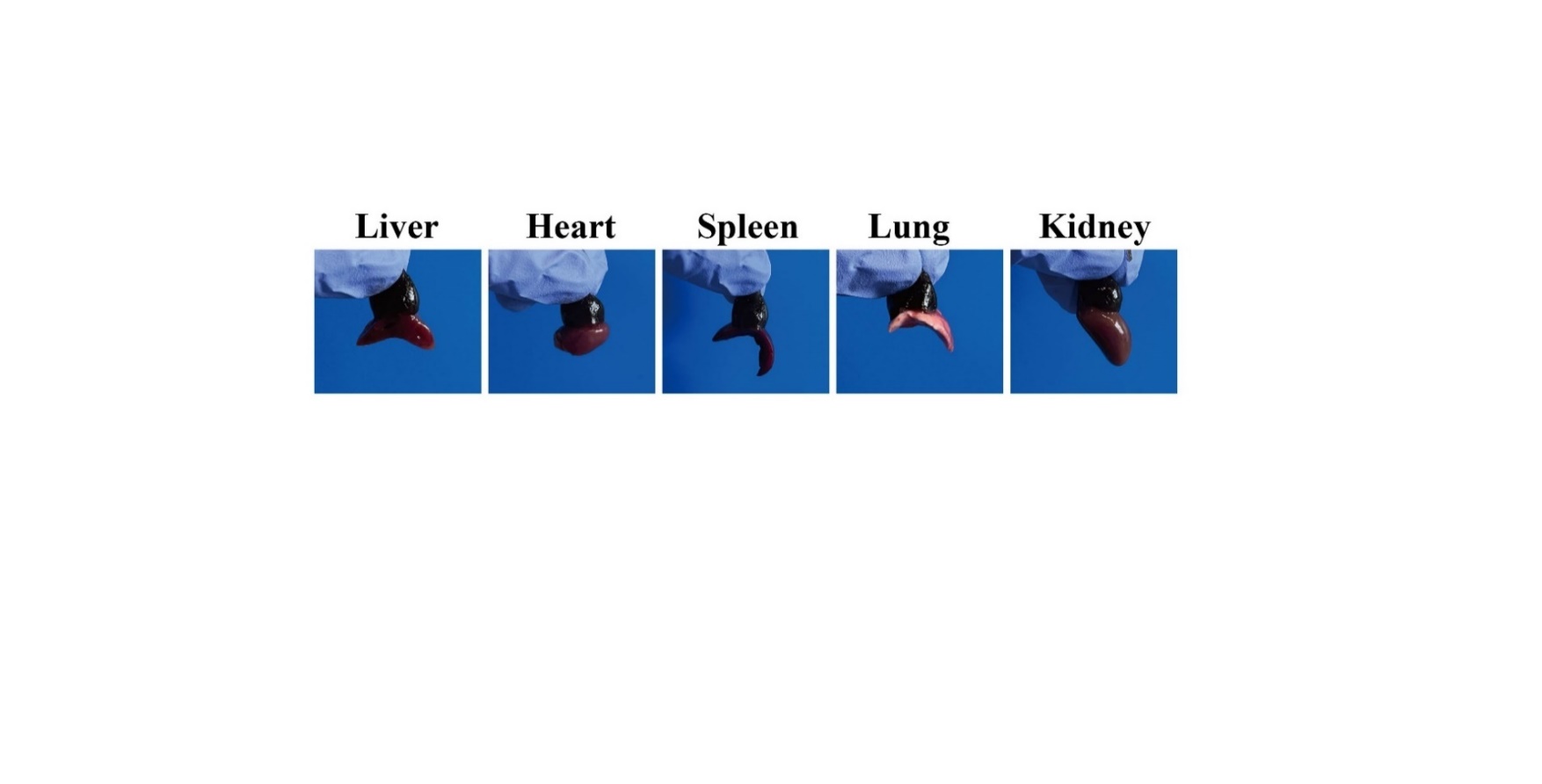


**Figure S10.** FSH3 hydrogel adhesion to different rat organs depicted.


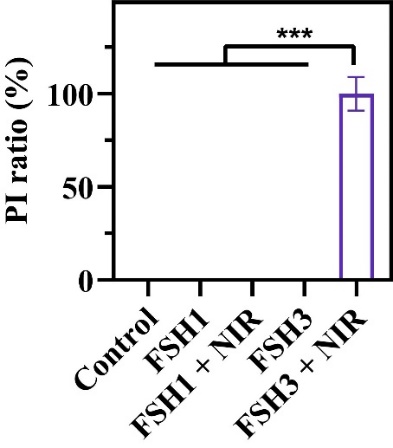


**Figure S11.** Comparison of PI fluorescence intensity in various experimental conditions for MRSA. Error bars correspond to mean ± SD (n = 3). *** signifies P < 0.001.


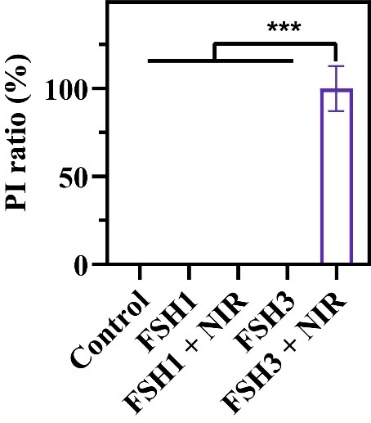
**Figure S12.** Comparison of PI fluorescence intensity across diverse experimental setups for MRPA. Error bars correspond to mean ± SD (n = 3). *** signifies P < 0.001.


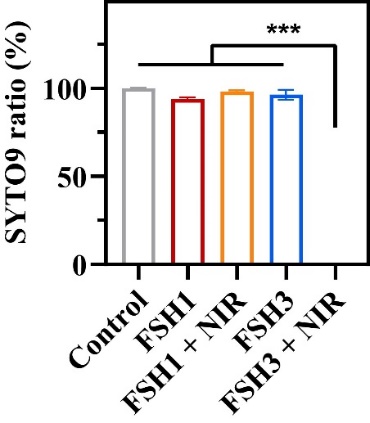


**Figure S13.** Comparison of SYTO9 fluorescence intensity in various experimental conditions for MRSA biofilm. Error bars r correspond to mean ± SD (n = 3). *** signifies P < 0.001.


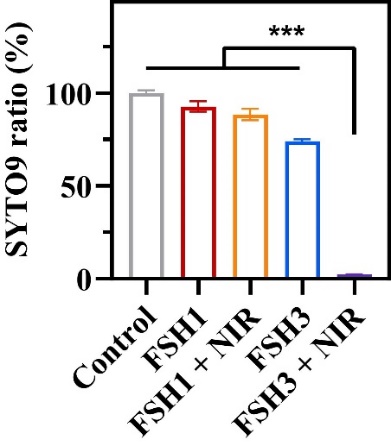


**Figure S14.** Comparison of SYTO9 fluorescence intensity across diverse experimental setups for MRPA biofilm. Error bars correspond to mean ± SD (n = 3). *** signifies P < 0.001.


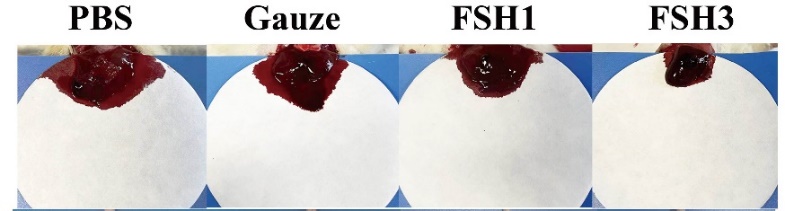


**Figure S15.** Macroscopic images showcasing the liver hemostasis model.


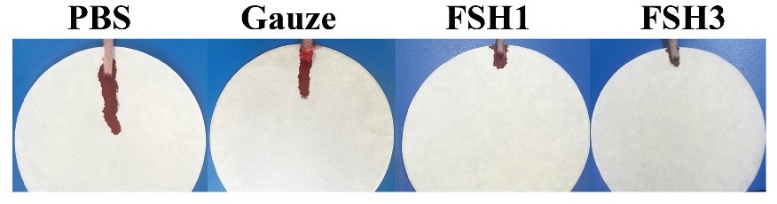
**Figure S16.** Macroscopic images of the tail hemostasis model.


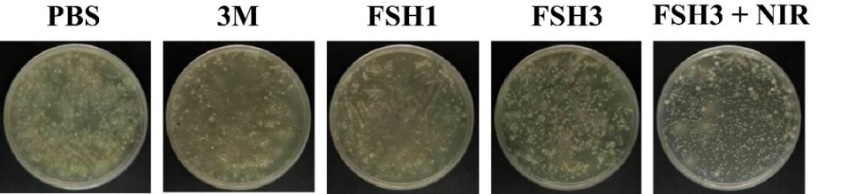
**Figure S17.** Images depicting bacterial colony development at rat wound sites.

**
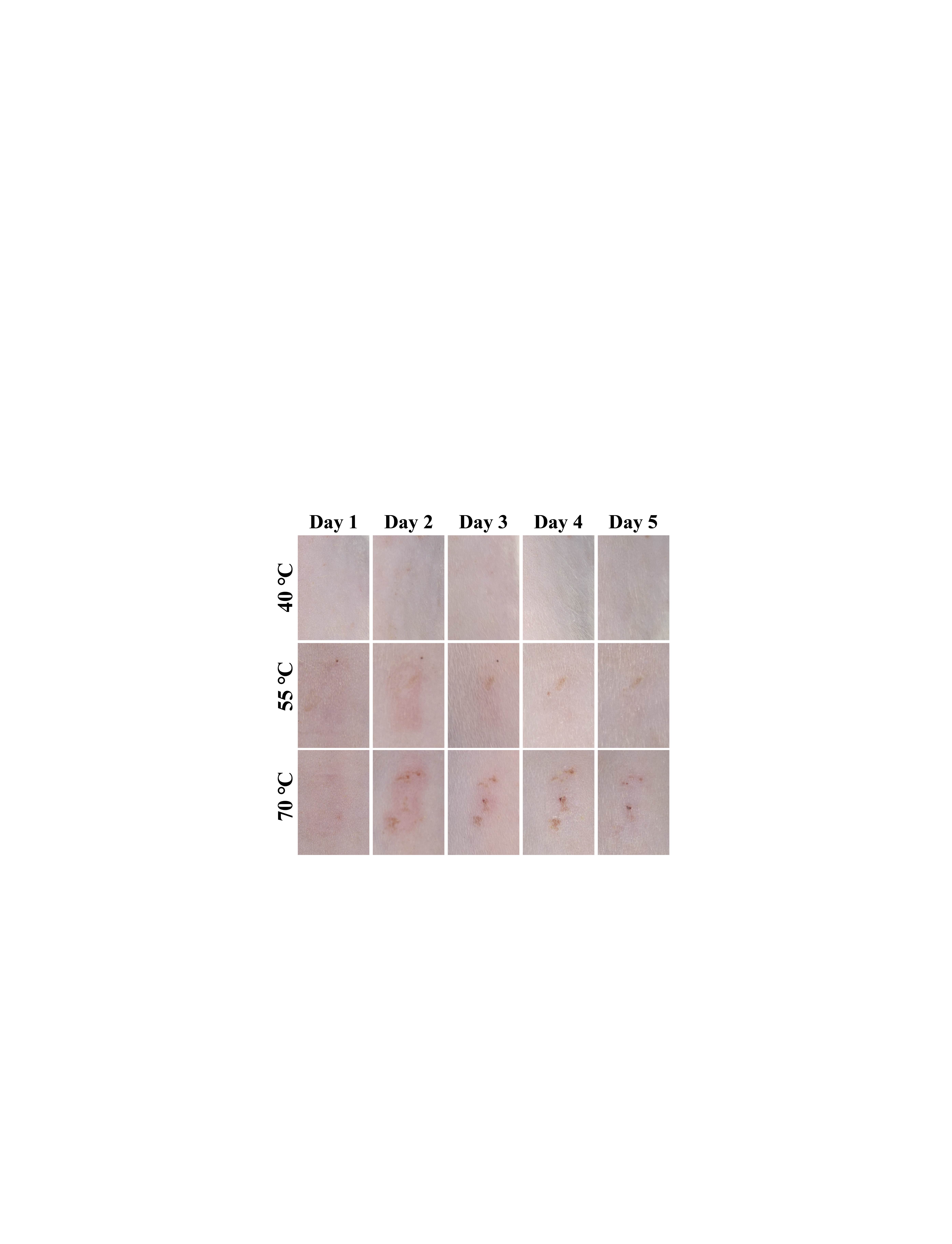
Figure S18.** Skin injury in rats exposed to temperatures of 40 °C, 55 ℃, and 70 ℃.


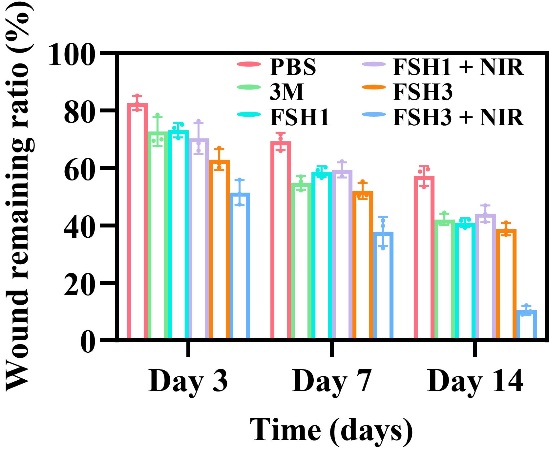


**Figure S19.** Comparison of wound dimensions across all groups. Error bars correspond to mean ± SD (n = 3).


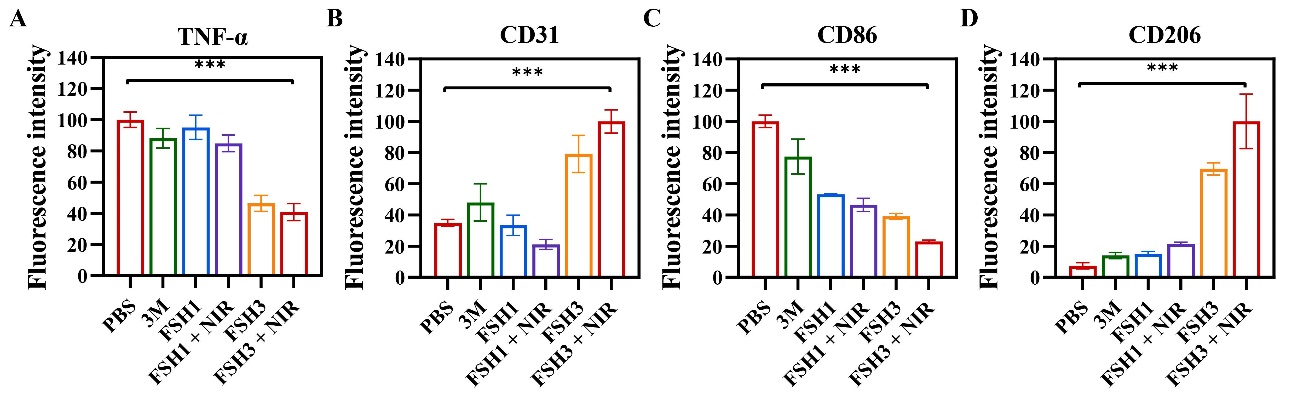
**Figure S20.** Analysis of fluorescence levels in tissue sections labeled with TNF-α (A), CD31 (B), CD86 (C), and CD206 (D). Error bars correspond to mean ± SD (n = 3). *** signifies P < 0.001.


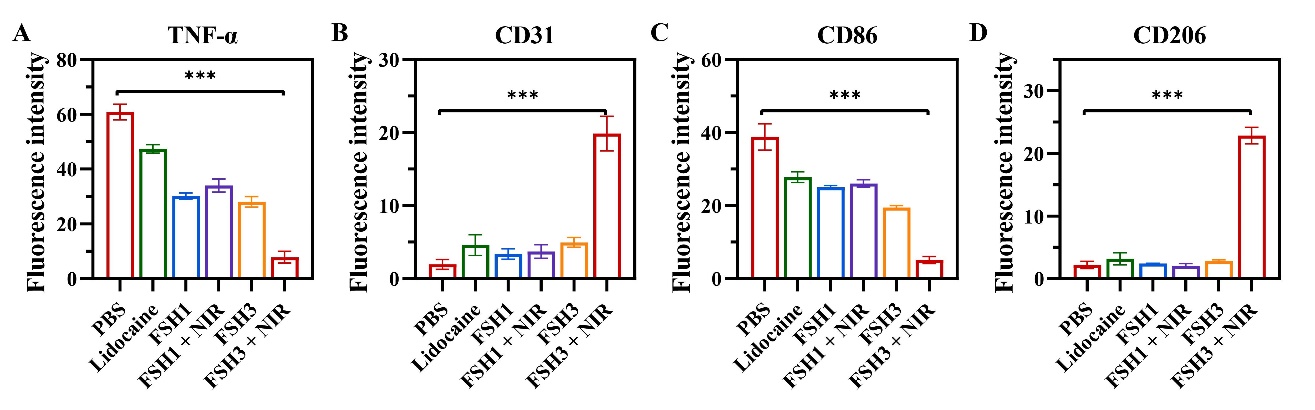
**Figure S21.** Analysis of fluorescence intensity in tissue sections marked with TNF-α (A), CD31 (B), CD86 (C), and CD206 (D). Error bars correspond to mean ± SD (n = 3). *** signifies P < 0.001.

**
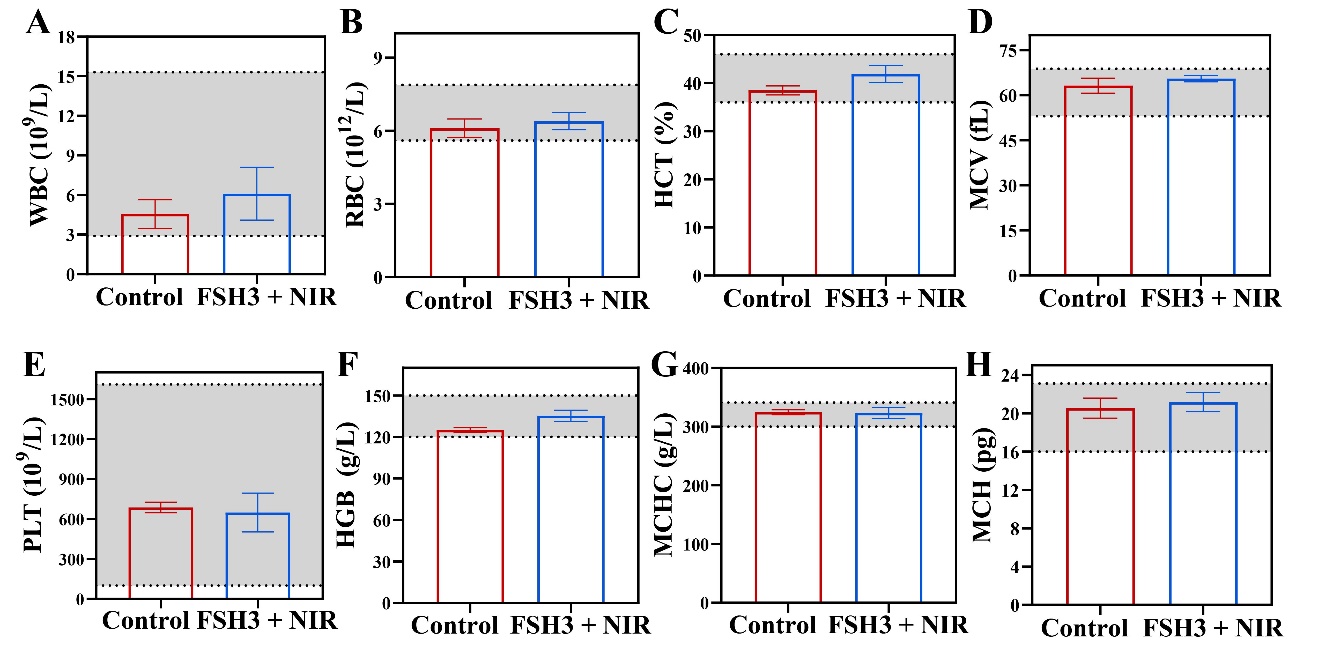
Figure S22.** Comprehensive blood analysis in diabetic rats treated with PBS or FSH3 + NIR on day 14, including white blood cell (WBC) (A), red blood cell (RBC) count (B), hemoglobin (HGB) (C), mean corpuscular volume (MCV) (D), platelet count (PLT) (E), hematocrit (HCT) (F), mean cell hemoglobin concentration (MCHC) (G), and mean corpuscular hemoglobin (MCH) (H), with error bars indicating mean ± SD (n = 3).


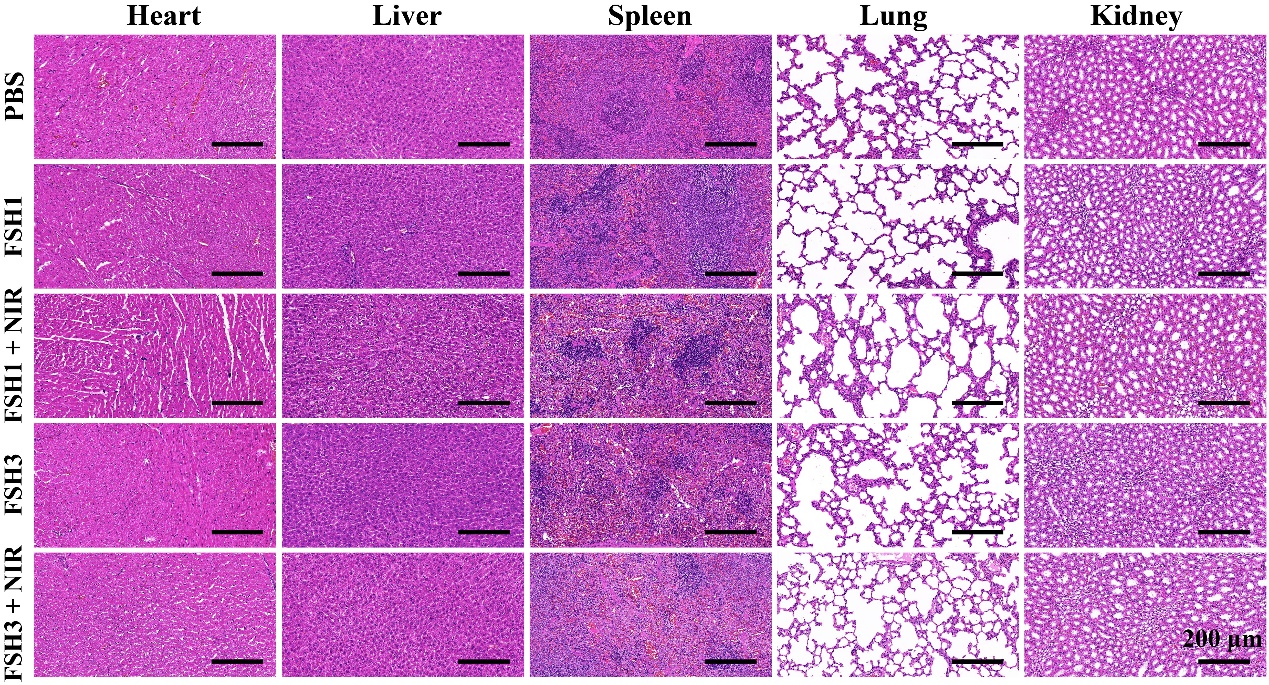
**Figure S23.** H&E staining of key organs in each group (scale bar: 200 µm).
